# Polyphenol Profile of *Cistus* × *incanus* L. and Its Relevance to Antioxidant Effect and α-Glucosidase Inhibition

**DOI:** 10.3390/antiox12030553

**Published:** 2023-02-22

**Authors:** Aneta Starzec, Maciej Włodarczyk, Dominika Kunachowicz, Andrzej Dryś, Marta Kepinska, Izabela Fecka

**Affiliations:** 1 Department of Pharmacognosy and Herbal Medicines, Faculty of Pharmacy, Wroclaw Medical University, Borowska 211a, 50-556 Wroclaw, Poland; 2 Department of Pharmaceutical Biochemistry, Division of Biomedical and Environmental Sciences, Faculty of Pharmacy, Wroclaw Medical University, Borowska 211a, 50-556 Wroclaw, Poland; 3 Department of Physical Chemistry and Biophysics, Faculty of Pharmacy, Wroclaw Medical University, Borowska 211a, 50-556 Wroclaw, Poland; 4 Committee for Therapeutics and Drug Sciences, Polish Academy of Sciences, Defilad 1, 00-901 Warsaw, Poland

**Keywords:** *Cistus* × *incanus*, polyphenols, flavonols, ellagitannins, antioxidants, α-glucosidase inhibition

## Abstract

The European Food Safety Authority recommends *C. incanus* as a natural source of antioxidants. Its activity is essentially determined by polyphenols, although specific compounds are not finally indicated. The available plant material comes from different subspecies and locations, which can lead to differences in chemical composition and potency. For this reason, we conducted a detailed analysis of the polyphenol content and antioxidant activity of 52 different *C. incanus* teas from Turkey, Albania, Greece, and unspecified regions. We focused special attention on ellagitannins, which have not been properly determined so far. Besides oxidative stress, hyperglycemia is an essential component of cardiometabolic diseases. Therefore, in subsequent experiments, we evaluated the ability of *C. incanus* extracts and individual polyphenols to inhibit α-glucosidase. Using statistical methods, we analyzed how differences in chemical composition affect activity. The results showed that *C. incanus* is a rich source of ellagitannins (2.5–19%), which dominate among polyphenols (5.5–23%). Turkish-origin products had higher ellagitannin content and a greater antioxidant effect (FRAP, ABTS) than Albanian and Greek products. In contrast, the flavonoid and phenolic acid contents and DPPH values were at similar levels in all products. An in-depth analysis of their composition indicated that all groups of polyphenols are involved in the antioxidant effect, but a significant contribution can be attributed to ellagitannins and flavonoids. *C. incanus* extracts showed a high capacity to inhibit α-glucosidase activity (IC_50_ 125–145 μg/mL). Ellagitannins were the most effective inhibitors (IC_50_ 0.7–1.1 μM), with a potency exceeding acarbose (3.3 mM). In conclusion, *C. incanus*, due to the presence of ellagitannins and flavonoids, exhibits powerful antioxidant and α-glucosidase inhibitory effects.

## 1. Introduction

*Cistus × incanus* L. is widely used in the food and pharmaceutical industries as an excellent source of antioxidants. Preparations of *C. incanus* have been recommended as a natural product providing compounds with such effects by the European Food Safety Authority (EFSA) since 2010 [1]. Its antioxidant activity is mainly associated with the presence of polyphenols.

The taxonomy of the *Cistus* (rock-rose) genus is complex, which can cause problems identifying individual species and plant material. Frequent polymorphism of vegetative and generative organs and possible hybridization between related species result in numerous subspecies being distinguished. For example, *C. incanus* is considered a hybrid between *C. albidus* and *C. crispus*. At present, we distinguish three subspecies: *incanus*, *corsicus*, and *creticus* (=*C. creticus*) [2,3,4]. Plant material from different subspecies and regions can vary significantly in chemical composition and activity.

Plants of the genus *Cistus* have been widely used in the traditional medicine of Mediterranean countries. Rock-rose has been recommended for skin wounds, respiratory inflammation, circulatory and urinary disease, and diabetes. Its extracts have also been used for gastrointestinal disorders, peptic ulcers, and diarrhea [4,5].

An imbalance between endogenous antioxidants and prooxidants is associated with the occurrence of oxidative stress and the production of multiple reactive oxygen species (ROS), which is strongly correlated with the pathogenesis of non-communicable chronic diseases. Polyphenols can quench ROS, chelate transition metals, trap reactive dicarbonyls (e.g., methylglyoxal), and, as a result, reduce lipid peroxidation and post-translational modification of proteins and lipoproteins, and protect DNA from interaction with a wide range of metabolites [6,7,8]. Therefore, *C. incanus* preparations are suggested as dietary supplements to strengthen the immune system and prevent many chronic diseases such as type 2 diabetes and cardiovascular, liver, and kidney diseases [4,6,7].

Type 2 diabetes is a severe chronic metabolic disorder that causes many health complications and is one of the leading causes of mortality [9,10]. Excessive postprandial hyperglycemia (PPHG) is one of the most important factors in the development of diabetes. PPHG is also a strong predictor of cardiovascular disease; it increases oxidative stress through vascular endothelial dysfunction and subclinical inflammation, resulting in atherosclerosis and cardiovascular events [10]. High post-meal glucose levels are closely related to the diet’s carbohydrate content and the action of α-glucosidase and α-amylase in the gastrointestinal tract. These enzymes are crucial for carbohydrate digestion [10,11]. In light of this, agents that can reduce PPHG by delaying the digestion of complex carbohydrates in the diet may be an attractive preventative option and an adjunctive treatment for diabetes [10]. There are scientific reports suggesting that some polyphenols may have the ability to inhibit carbohydrate hydrolases, but there are few such studies on *Cistus* extracts [11].

Rock-rose is characterized by a wide variety of polyphenols. Despite the availability of literature data, its complete chemical profile is not yet known. The contents of individual components depend on genotype, environmental, cultivation, and storage conditions [4,12]. The main compounds found in *C. incanus* include flavonoids; among them, flavonols and flavan-3-ols are distinctive [13,14,15,16,17]. The non-flavonoid compounds include ellagitannins [15,18,19]. In addition, free phenolic acids are present [17,20]. All *Cistus* species also contain essential oil and a brown resin comprising labdane-type diterpenes [13,14,16].

In a study evaluating the effect of regular administration of *C. incanus*, Kuchta et al. [21] observed a reduction in cardiometabolic risk factors in healthy volunteers, including oxidative stress and dyslipidemia, by improving the lipid profile. Moreover, our previous in vitro studies showed that rock-rose flavonols exhibit antiglycative activity by inhibiting protein glycation [8]. However, the components responsible for the observed antioxidant, antidiabetic, and hypolipidemic effects have not been identified, while the market of *Cistus* products of different or unspecified origins is growing.

Therefore, we aimed to compare the antioxidant activity in relation to the chemical composition of *C. incanus* teas of different origins. We evaluated the content of polyphenols, including individual ellagitannins, flavonoids, and phenolic acids. Furthermore, in our study, we assessed in vitro the potential of *C. incanus* to modify polysaccharide metabolism through the effects on α-glucosidase by analysis of both the extracts themselves and individual polyphenols. Using statistical methods, we investigated how the chemical profile and its revealed differences affect antioxidant and anti-glucosidase activity.

## 2. Materials and Methods

### 2.1. Chemicals and Reference Materials

Folin–Ciocalteu reagent, iron (III) chloride, and iron (II) sulfate heptahydrate were obtained from Chempur (Poland). Aluminium chloride and 2,4,6-tris-(2-pyridyl)-*s*-triazine (TPTZ) was from Fluka (Switzerland). ABTS, DPPH, DMSO, Trolox, acarbose, 4-nitrophenol (*p*-NP), 4-nitrophenyl-α-D-glucopyranoside (*p*-NPG), α-glucosidase from *Saccharomyces cerevisiae*, LC and LC-MS grade solvents, and others were from Sigma-Aldrich (Germany).

Myricetin, myricetin-3-*O*-α-rhamnoside (myricitrin), myricetin-3-*O*-β-galactoside (gmelinoside I), quercetin, quercetin-3-*O*-α-rhamnoside (quercitrin), quercetin-3-*O*-β-glucoside (isoquercitrin), quercetin-3-*O*-β-galactoside (hyperoside), quercetin-3-*O*-β-glucuronoside, kaempferol, kaempferol-3-*O*-β-glucoside (astragalin), kaempferol-3-*O*-β-glucuronoside, kaempferol-3-*O*-β-(6″-*O*-*p*-coumaroyl)glucoside (tiliroside), gallic acid, ellagic acid, (+)-catechin, (−)-epicatechin, procyanidins A2, B1, B2 and C2 were purchased from Extrasynthese (France). Cistusin, punicalagin, and terflavin A were isolated from *C. incanus* by Fecka et al. [18], and myricetin-3-*O*-β-glucuronoside by Fecka [22]. All assayed compounds were at least of 95% purity (HPLC-DAD).

Stock solutions (1 mg/mL) were prepared with methanol for most compounds and with DMSO for ellagic acid. The obtained solutions were then diluted with 50% aq. methanol (*v*/*v*) to concentrations 20–200 μg/mL, and filtered by Durapore 0.45 and 0.22 μm filters (Millipore, Sigma-Aldrich).

Some minor polyphenols were identified by co-chromatography with the reference materials (Galke, Germany): helichrysoside → *Helichrysum arenarium* [23,24], kaempferol-3-*O*-β-galactoside → *Menyanthes trifoliata* [25], myricetin-3-*O*-β-glucoside → *Humulus lupulus* and *Vitis vinifera* [26,27], myricetin-3-*O*-α-arabinofuranoside → *Polygonum aviculare* [28], quercetin-3-*O*-β-xyloside → *Malus domestica* and *Pyrus communis* [29], quercetin-3-*O*-α-arabinofuranoside → *Polygonum aviculare* [28], punicalin → *Punica granatum* [30].

### 2.2. General Equipment

LC analysis was performed in a Dionex Ultimate 3000 liquid chromatograph with an autosampler (WPS-3000TSL), a pump (LPG-3400SD), a column thermostat (TCC-3000SD), and a diode detector (DAD-3000) (Thermo-Fisher Scientific, Sunnyvale, CA, USA). The system was connected with Chromeleon 7 software.

Spectrophotometric measurements were conducted using a Multiskan GO Spectrophotometer (Thermo-Fisher Scientific). The %Inhibition was calculated from the formula

$$\%\;\mathrm{inhibition}=\left(\frac{A_{\mathrm{Control}}-{\;A}_{\mathrm{Sample}}}{A_{\mathrm{Control}}}\;\right)\times 100$$

MS spectra in the negative mode were recorded on an ESI-qTOF Compact mass spectrometer (Bruker, Bremen, Germany). The mass spectrometer was re-calibrated for every run [31]. ^1^H-NMR, ^13^C-NMR, HSQC, HMBC, and COSY experiments were recorded on Avance 300 MHz spectrometer (Bruker) in DMSO-*d6* and calibrated using the residual solvent peak. The data were processed with MestReNova 12 software (Mestrelab Research, Santiago de Compostela, A Coruña, Spain).

### 2.3. Plant Material and Extracts

The material used in this study consisted of 52 commercial products (teas) containing dried leaves or shoot tops (herbs) of *Cistus* × *incanus* L. (labeled as Ci1–Ci52). The products were purchased from various companies and came from different countries (Albania, Greece, Turkey) or had no specific origin. All *C. incanus* samples (approx. 10 g each) were homogenized with an analytical mill A 11 basic (IKA, Königswinter, Germany) and sieved through a 0.355 mm sieve (Multiserw-Morek, Marcyporęba, Poland).

Approx. 1 g of homogenized material was transferred into 50 mL of 55% aq. methanol (*v*/*v*) and heated in a water bath under a reflux condenser for 15 min after reaching the boiling point. Next, the extracts were decanted and filtered through Durapore 0.45 and 0.22 μm filters. The drug-extract ratio was 1:50. Extracts were prepared in triplicate.

### 2.4. Preparative Separation of C. Incanus Flavonols

The main flavonols were isolated from *C. incanus* water-acetone extract on octadecyl by column chromatography and then purified on Sephadex LH-20. Mixtures of water-methanol (RP-18) and methanol (LH-20) were used as eluents. The procedure was described previously [18].

The resulting compounds were subjected to MS and NMR. Spectroscopic data were compared with data from the scientific literature and authentic standards.

### 2.5. UHPLC-ESI-qTOF-MS and HPLC-DAD Experiments

In general, the conditions for confirming the analyzed polyphenols’ identity were described before [18,31]. The separations were achieved on the Ultimate 3000 liquid chromatograph with DAD and ESI-qTOF Compact detectors and an octadecyl Kinetex column (150 × 2.1 mm; 2.6 μm; Phenomenex, Torrance, CA, USA). The resulting chromatograms, together with base and fragmentation mass spectra, were analyzed with Data Analysis 4.2 software (Bruker).

The content of polyphenols was analyzed on an octadecyl Hypersil Gold column (250 × 4.6 mm; 5 μm; Thermo-Fisher Scientific) by the method described previously [18]. Flavonols, ellagic acid and ellagitannins were detected at 254 nm, gallic acid and flavan-3-ols at 280 nm, and coumaroyl-flavonols at 320 nm (Table S5). For each polyphenol, the mean content in the test product and the standard deviation (SD) were determined. In addition, the mean compound content, SD, median, minimum, and maximum values were calculated for the groups of products. Due to the lack of authentic standards, some components were calculated from regression equations of related compounds. The amount of hexahydroxydiphenoyl-glucose and punicalin was calculated as punicalagin, myricetin-3-*O*-glucoside and myricetin-3-*O*-arabinoside as myricetin-3-*O*-galactoside, quercetin-*O*-arabinoside as quercetin-3-*O*-galactoside, helichrysoside and coumaroyl-tiliroside as tiliroside. Differences in molecular masses were taken into account in each conversion. Sums of ellagitannins (SET), phenolic acids (SPA), flavonoids (SF, flavonols+flavan-3-ols), and polyphenols (SPP) were obtained by summing the compounds in each group.

### 2.6. Total Phenolic (TPC) and Flavonoid (TFC) Content 

TPC was estimated using the Folin–Ciocalteu method described by Singleton et al. [32], while TFC was estimated according to the European Pharmacopoeia method for *Betulae folium* [33], with some modifications. A detailed description of the procedures can be found in our publication [8]. TPC results were expressed as mg of gallic acid equivalents per gram of dry weight (mg GAE/g d.w.). The TFC was expressed as myricetin equivalents (mg ME/g d.w.).

### 2.7. Antioxidant Activity Tests

#### 2.7.1. DPPH and ABTS

The modified Blois method was used [34]. In a 96-well microplate, 200 µL of DPPH solution was added to 20 µL of each diluted extract (1:30). The plate was incubated for 30 min at ambient temperature in the dark. The absorbance was measured at 517 nm.

The method developed by Chen and Kang [35] was applied to evaluate the antioxidant effect using the ABTS radical. ABTS reagent was obtained according to the procedure described previously [8]. In a 96-well microplate, 200 µL of ABTS reagent and 2 µL of each diluted extract (1:30) were mixed. The plate was incubated for 15 min. Absorbance was measured at 734 nm.

Calibration was set for gallic acid (0.06–0.6 µM/mL). Results were expressed as a percentage of DPPH or ABTS radical inhibition and as mM of gallic acid equivalents per gram of dry weight (GAE/g d.w.).

#### 2.7.2. FRAP

The FRAP assay, according to the method of Benzie and Strain [36] with slight modifications [8], was used. Then, 20 µL of diluted extracts (1:30) and 200 µL of FRAP reagent were each applied to the microplate. The plate was incubated for 4 min at ambient temperature in the dark. Absorbances at 593 nm were measured.

Calibration was performed for both ferrous ions Fe(II) (0.01–0.6 µM/mL) and gallic acid (0.1–2 µM/mL). Results were expressed as mM of Fe(II) per gram of dry weight and mM of GAE/g d.w.

### 2.8. α-Glucosidase Inhibitory Assay

The α-glucosidase inhibitory activity was conducted in a 96-well microplate using 4-nitrophenyl-α-D-glucopyranoside (*p*-NPG), as reported previously [37]. Briefly, 90 µL of 0.1 M sodium phosphate buffer (pH = 6.9) was mixed with 50 µL of sample (250 µg/mL, methanol-buffer 1:1) and 40 µL of enzyme solution (0.25 U/mL). Acarbose (5 mg/mL) was used as a positive control. The microplate with samples and enzyme solution was initially incubated for 5 min at 37 °C. After preincubation, the reaction was started by adding 20 µL of 5 mM *p*-NPG to the wells containing extracts/polyphenols or acarbose. The reaction was allowed to run for 10 min and afterward stopped by adding 50 µL of 0.1 M Na_2_CO_3_. The absorbance was measured at 405 nm. All of the samples were tested in triplicate.

The readings were reduced by absorbance of the sample blanks measured separately for each extract/polyphenol, which consisted of 50 µL of extract diluted in 150 µL of buffer solution only, while values obtained for uninhibited controls were reduced by absorbance values of the buffer solution (buffer blank). The activity was expressed as %inhibition. The half-maximal inhibitory concentration (IC_50_) values, defined as concentration of inhibitor necessary to reduce the rate of an enzymatic process by 50%, were calculated as follows:

$${\mathrm{IC}}_{50}=\frac{\mathrm{sample}\;\mathrm{concentration}\;\times 50}{\%\;\mathrm{inhibition}}$$

### 2.9. Kinetics of α-Glucosidase Inhibition 

Polyphenols with >70% of α-glucosidase inhibition (gallic acid, ellagic acid, cistusin, punicalagin, terflavin A, tiliroside) were subjected to a kinetic study aiming to determine their mode of enzyme inhibition. Inhibition modes of α-glucosidase were determined according to Chu et al. [38]. Basically, the varying concentrations of polyphenols (0.5–250 µg/mL) and acarbose (2.5–10 mg/mL) were tested for inhibitory activity in the range of increasing *p*-NPG concentrations (1–20 mM) with a fixed enzyme concentration (0.25 U/mL). After 5 min of preincubation, the addition of the substrate started the reaction, and the changes in absorbance were measured at 30 s intervals. The uninhibited reaction was also run. The reaction was terminated after 10 min, and the absorbance values obtained in the kinetic study were used to create Lineweaver–Burk plots of 1/*v* against 1/[S].

### 2.10. Statistical Analysis

Statistica software (StatSoft, TIBCO Software Inc., Palo Alto, CA, USA) was used for statistical analysis. Normality of distribution was checked using the Shapiro–Wilk test. Analysis of variance (ANOVA) and post-hoc Fisher’s LSD test was performed. Correlation between data was checked using linear regression and Spearman’s rank correlation coefficient. Statistical significance for all tests was taken as *p* < 0.05. For details, see the Supplementary Materials in Section S4.

## 3. Results and Discussion

*C. incanus* teas are popular as products with antioxidant potential. Therefore, they are used in the prevention and supportive therapy of many chronic diseases, including cardiometabolic diseases, in which oxidative stress and hyperglycemia are relevant to pathomechanism [5,6,7,16,21,39].

Rock-rose is also a source of many polyphenols, but their profile has not been fully defined, especially for materials of diverse origins [5,13,15,16,18,20,21]. Therefore, we carried out a detailed study of the polyphenol profile in 52 commercial *C. incanus* teas, of which 23 were of Turkish origin, 10 were from Albania, and 3 were from Greece (the data obtained for these materials may not be precise due to the small number of samples). The other products had no specified place of cultivation. However, our previous study showed that these products were possibly Albanian [8]. The present work is a continuation of that research.

Since *C. incanus* is well described in terms of antioxidant properties, we conducted antioxidant tests to compare the effect of the chemical profile on this action. It is likely that one of the groups of rock-rose polyphenols is ellagitannins, as described previously [18], so we focused on the determination of individual compounds from this group (punicalagin, cistusin, terflavin A). Flavonoids and phenolic acids were also quantified. In the first stage, we standardized the extraction method. Comprehensive information can be found in the Supplementary Materials (Section S1, Tables S1–S4). Extraction with 55% aq. methanol was chosen for routine assays. Then, the content of polyphenols determined by chromatographic and spectrophotometric methods was compared. Since punicalagin is a known inhibitor of α-glucosidase [40], we decided to test *C. incanus* extracts and individual polyphenols as potential inhibitors of an enzyme involved in polysaccharide metabolism.

### 3.1. C. Incanus Polyphenols

The polyphenol profile was verified by UHPLC-ESI-qTOF-MS using extracts from six products (Ci5, Ci30, Ci38, Ci47, Ci48, Ci53) of different origins. In addition, two solvent gradients were used to enhance the information on co-eluting compounds. For polyphenol identification, authentic standards, ellagitannins, and flavonoids isolated from *C. incanus* (Section 2.4) were used. Details of the identified compounds are summarized in Table S5.

UHPLC-ESI-qTOF-MS analysis identified 54 polyphenolic compounds (peaks 1–54) based on reference substances, literature data, interpretation of UV/Vis spectra, retention times, and mass spectra. Among the tannins and related compounds were cistusin, punicalagin, punicalin, terflavin A, hexahydrodiphenoyl-glucose (HHDP-Glc), gallic and ellagic acids, ellagic acid glycosides, as well as flavan-3-ols such as catechin, epicatechin, and gallocatechin.

We isolated the main ellagitannins and flavonoids according to the procedure described before [18]. The following common flavonols were identified by HRMS and MS/MS together with NMR: myricetin-3-*O*-β-galactoside (=gmelinoside-I), myricetin-3-*O*-β-rhamnoside (=myricitrin) and myricetin-3-*O*-α-arabinoside. Of them, myricetin-3-*O*-β-galactoside was isolated by Gürbuz et al. [41] from *C. salviifolius* and reported by Riehle et al. [42] in *C. incanus*, myricetin-3-*O*-α-arabinoside was not confirmed in *C. incanus* previously while myricetin-3-*O*-β-rhamnoside was reported in the genus *Cistus* by Wittpahl et al. [15]. The other observed compounds with a myricetin core were myricetin-*O*-hexoside gallate and minor pentoside. The latest one was co-chromatographed with *Polygonum aviculare* herb extract and identified as myricetin-3-O-α-arabinoside (=betmidin, furanoside form) [28].

The main quercetin glycosides—3-*O*-β-galactoside (=hyperoside), 3-*O*-β-glucoside (=isoquercitrin), and 3-*O*-β-rhamnoside (=quercitrin)—from fractions were additionally compared with authentic standards. Of them, hyperoside was isolated by Gürbuz et al. [41] from *C. salviifolius* and reported by Wittpahl et al. [15] and Riehle et al. [42]. Among quercetin pentosides, the main isomer was presumed as 3-α-L-arabinopyranoside due to its highest intensity in this group and a previous report of this compound [41], and above its MS/MS typical for 3-*O*-substituted flavonols; quercetin-*O*-pentoside at 12.10 min with MS/MS in the manner of 3-*O*-substituted flavonols was presumed to be 3-*O*-xyloside of quercetin, following co-chromatography with its known source, i.e., pear and apple skin extracts [29], while the slower isomer at 12.35 min was confirmed to be avicularin (quercetin-3-α-L-arabinofuranoside) by co-chromatography with *Polygonum aviculare* extract [28]. Still, the amount was insufficient to perform an NMR analysis. Similarly to myricetin, tentatively identified quercetin-*O*-hexoside gallate was observed at 11.28 min. The following two compounds were previously reported in the genus *Cistus*: CAS: 1891055-64-3 and quercetin-3-*O*-α-arabinopyranoside in *C. salviifolius* [41].

Concerning kaempferol glycosides, two *O*-hexosides were observed, both giving MS/MS fragments typical for 3-*O*-substituted flavonols. The second, at 12.42 min, was confirmed as astragalin with the authentic standard, while the first, at 12.19 min, was confirmed to be trifolin (=kaempferol-3-*O*-β-D-galactopyranoside) by co-chromatography with *Menyanthes trifoliata* extract [25]. Previously, the sugars for kaempferol glycosides were attributed by Wittpahl et al. [15] tentatively only. A minor compound at 417 *m/z* was identified as kaempferol-3-*O*-pentoside.

The last distinctive group of flavonols comprised esters with coumaric acid. The most intense ions originated from a pair of *E/Z*-tilirosides (denoted in Table S5 as its isomers b and c, confirmed with the authentic standard) accompanied by a more rapidly eluting small one (probably 7-*O*-substituted kaempferol-6″-coumaroylglucoside, e.g., buddlenoid A) or tiliroside analog with galactose core instead of glucose. Three additional small molecules corresponding to coumaroyl esters of tiliroside were observed at the end of the chromatogram. Coumaroyl-tiliroside was previously described by Wittpahl et al. [15]. On the chromatogram, tiliroside was preceded by three molecules with 609 *m/z* and a fragmentation pattern analogous to tiliroside, except that the core molecule (MS/MS) was quercetin or its analog. Such compounds were helichrysoside (confirmed by co-chromatography) with *Helichrysum arenarium* extract [24] and its isomers, not previously reported in rock-rose.

All ellagitannins appeared on UHPLC chromatograms as two peaks each, most likely according to an equilibrium of α/β anomers of the central sugar. Other known polyphenols such as gallic acid, ellagic acid, catechin, and epicatechin were also identified compared to authentic standards. Additionally, minor amounts of glycosides such as ellagic acid pentosides, hexoside, and deoxyhexoside were observed in the extracts.

The used MS conditions were insufficient to observe and identify procyanidins that were reported previously [43]. It may be that other polyphenols present in larger quantities hindered the analysis. A different assay or extraction technique would have to be used to obtain the procyanidin fractions, which requires further experiments.

### 3.2. Quantification of Polyphenols, Flavonoids, Tannins, and Phenolic Acids in C. Incanus

In most scientific works, the total phenolic content (TPC) was determined by the Folin–Ciocalteu method; therefore, we initially determined the TPC of extracts using this method. In addition, the total flavonoid content (TFC) expressed as myricetin equivalents (ME) was determined. The same extracts were then subjected to quantitative analysis by HPLC-DAD. Detailed data (mean, SD, median, minimum, and maximum values) for products of different origins are shown in Table 1. It also includes the sum of polyphenols, flavonoids, ellagitannins, and phenolic acids determined by HPLC-DAD, while the content of individual polyphenolic compounds is shown in Table 2.

#### 3.2.1. Quantification of TPC and TFC

Total phenolics in *C. incanus* extracts are presented as gallic acid equivalents per gram of dry weight of the product. The mean TPC for all samples (Ci1-Ci52) was estimated at 350 mg GAE/g d.w. (range 14–623 mg/g). Samples of Turkish and Albanian origin had similar TPC, 354 mg/g and 328 mg/g, respectively. A high content was recorded in samples from Greece, with 403 mg/g. However, the differences were not statistically significant (Table S10). Slightly lower TPC values (269–347 mg/g) were recorded in the study of Gaweł-Bęben et al. [6] in 60% hydromethanolic and methanolic extracts. In 80% hydromethanolic extracts of a dozen *C. incanus* products of different origins, TPC ranged from 2 to 148 mg GAE/g d.w. As in our study, it was noted that products of Turkish origin had the highest content of polyphenols (84 mg/g), especially compared to products of unknown origin, which were the poorest in these compounds [20]. Lower TPC was also obtained for 50% [44] and 30% [45] hydroethanolic extracts, where the amount of polyphenols was 42–99 mg and 36–89 mg GAE/g d.w., respectively.

In addition to the high TPC content, samples of Turkish origin had the highest TFC (mean 45 mg ME/g d.w.) compared to Greek and Albanian origin (mean 40 and 33 mg/g, respectively). The TFC for all samples tested averaged 40 mg/g and ranged from 21 to 72 mg/g. In other studies, TFC values, converted to the selected flavonol, e.g., quercetin equivalents (QE) or rutin equivalents (RUE), were significantly lower than the results expressed as ME. Similar TFC values to those in the present study, expressed as quercetin, were obtained by Gaweł-Bęben et al. [6] (45–54 mg QE mg/g d.w.). A higher flavonoid content calculated as quercetin 41–138 mg QE mg/g d.w. was obtained in another study [45]. The TFC expressed as rutin was 11 mg (RUE/g d.w.) [46].

#### 3.2.2. Quantification of Polyphenols, Flavonoids, Tannins, and Phenolic Acids by HPLC-DAD

Analyzing the mean contents of individual polyphenols in the *C. incanus* products, the largest amounts were recorded for ellagitannins, 73 mg/g d.w. (SET range 26–189 mg/g). Turkish products had the highest SET content (94 mg/g) compared to the other countries, Greece (64 mg/g) and Albania (52 mg/g). The differences were statistically significant (Tables S10 and S11, Figure S3). A total of five compounds belonging to this group were determined, of which punicalagin was the main one with an average value of 29 mg/g d.w., followed by cistusin with 25 mg/g and terflavin A with 14 mg/g. In addition, the hexahydroxydiphenoyl-glucose isomers previously mentioned by Wittpahl et al. [15] were detected. Their amount was six times lower than that of punicalagin, with a mean 5 mg/g d.w. (range 0.7–13 mg/g). Furthermore, small amounts of punicalin were reported (mean 0.4 mg/g). It was not possible to determine proanthocyanidins because their content was below the LOD (Table S7).

The mean phenolic acid content of *C. incanus* teas was estimated at ~10–11 mg/g d.w. (SPA range 5–21 mg/g). Among these, only gallic and ellagic acids were present in higher amounts, averaging 7 and 3 mg/g, respectively. Both compounds were found in similar amounts in products of different origins.

The third group consisted of flavonoids, in which monomeric flavan-3-ols (epicatechin and catechin) and flavonols (glycosides of myricetin, quercetin, and kaempferol) were recorded. It was consistent with previous reports [13,14,15,16,17,20]. The sum of flavonoids (SF, flavonols+flavan-3-ols) was determined to be 18 mg/g d.w. (range 12–23 mg/g). There were no statistically significant differences between products of various origins. Flavan-3-ols occurred at similar levels (1 mg/g) in the 0.4–2.4 mg/g. Products from different origins contained comparable amounts of catechin and epicatechin. *C. incanus* contained flavonol monoglycosides with sugars such as galactose, glucose, rhamnose, arabinose, and xylose. Larger amounts were recorded for rhamnosides and galactosides, i.e., myricitrin (mean 4 mg/g), hyperoside (mean 3 mg/g), myricetin-3-*O*-galactoside (below 3 mg/g), and coumaroyl-flavonols such as tiliroside (mean 2 mg/g). In addition, myricetin-3-*O*-arabinoside, myricetin-3-*O*-glucoside, quercetin-*O*-arabinoside, quercitrin, and kaempferol-3-*O*-glucoside were detected in smaller amounts. Other coumaroyl-flavonols were also detected in relatively large amounts—coumaroyl-tiliroside (mean 1 mg/g) and helichrysoside (mean 0.4 mg/g). The flavonoid content estimated by HPLC-DAD vs. spectrophotometric methods was ~2 times lower but reached the same order of magnitude (18 vs. 40 mg/g d.w., respectively). The ratio of TFC:SF varied around a value of 1.2–4 (median 2). It is believed that using an appropriately selected standard (myricetin) gives more realistic results than using rutin or quercetin, which were used in other studies, and the calculated flavonoid content was lower [6,20,46].

The sum of polyphenols (SPP), obtained by adding the determined contents for the individual compounds, in all *C. incanus* products was estimated at 102 mg/g d.w. (range 54–229 mg/g), and the highest polyphenol sum was found in Turkish (124 mg/g), followed by Greek (92 mg/g) and Albanian (80 mg/g) products. The differences were statistically significant (Tables S10 and S11, Figure S3). SPP was more than three times lower compared to TPC. The ratio of polyphenol content for both methods ranged from 0.2 to 10 (median 3). The spectrophotometric method is used for the approximate estimation of polyphenols, and its results may be overestimated due to the presence of other substances in the extracts that can react with the Folin–Ciocalteu reagent. Chromatographic methods, on the other hand, allow quantitative analysis of individual polyphenols, which improves accuracy, especially if the appropriate profile of compounds is used as reference substances. In the statistical analysis, we detected a correlation between the results from the spectrophotometry and HPLC-DAD, which means that they reflect the difference in the content of components but do not accurately report their quantity. 

In Ci1-Ci52 products, ellagitannins accounted for the majority of all polyphenols at ~69% (range 48–85%, median 69%), followed by flavonoids at ~20% (range 8–41%, median 18%) and phenolic acids at ~11% (range 5–16%, median 12%). In products of Turkish origin, the ellagitannin content was the highest compared to the other countries, reaching 73% (Greece 68%, Albania 63%). In these products, the greatest difference was also noted between ellagitannins and flavonoids, with five times lower content (17%). In products from other countries, the percentage of flavonoids in SPP was higher, at 23% for Albanian and 21% for Greek products. Albanian products had the highest proportion of phenolic acids (14%), followed by Greek (11%) and Turkish (10%) products. Most of the polyphenols determined by the chromatographic method were found in the highest amount among teas of Turkish origin (12 out of 21 compounds). Detailed data on their content can be found in the Supplementary Material in Tables S6 and S8.

There are only a few works in the scientific literature dedicated to the content of individual polyphenols in *C. incanus*. Wittpahl et al. [15] analyzed 50% hydromethanolic extracts of four products. The sum of polyphenols in this study was 9–30 mg/g d.w., a significantly lower result than ours. The sum of ellagitannins (per gallic acid) in the German study ranged from 4 to 15 mg/g, and the dominant representative of this group was punicalagin (~4 mg/g). Nevertheless, these authors did not use authentic standards of ellagitannins. In the current work, the sum of ellagitannin significantly exceeded the results of the data cited above, but it is worth noting that punicalagin was also present in the highest amount (29 mg/g). The situation is similar for flavonoids. Results of Wittpahl et al. [15] ranged from 5 to 15 mg/g d.w. per rutin, with myricitrin in the highest amount (~4 mg/g). Our sum of flavonoids is slightly higher, while an identical amount of myricitrin was recorded. It should be noted that we determined a higher number of flavonoids. Their content, and simple glycosides, are influenced by the abundance of coumaroyl-flavonols, such as tiliroside and its analogs.

Viapiana et al. [20] determined 14 polyphenolic compounds (including flavonoids and phenolic acids) in hydromethanolic extracts but in lower amounts. The authors emphasize that higher contents of active compounds are found in products of Turkish and Albanian origin compared to products from Cyprus or of unknown origin. The results of the present study are significantly greater, while a similar correlation was noted between the content of polyphenols and the country of origin of the product.

The determination of polyphenol content is influenced by the choice of the test method, the parameters used, the apparatus settings, and the standard substances used. In addition, a very important factor influencing the content of polyphenolic compounds is the raw material itself—this mainly concerns environmental factors (place of origin, time of harvest, etc.) and factors related to the preparation of the commercial product (e.g., storage conditions, particle size, the ratio of leaf and stem content) [12,42,44,45,47].

### 3.3. In Vitro Antioxidant Potential of C. incanus

Frequently used methods to measure antioxidant activity include DPPH, ABTS, or FRAP tests. These methods work according to the single electron transfer mechanism; however, the principle of operation is different [34,35,36]. The results of *C. incanus* antioxidant activity, by country of origin, obtained in this study are shown in Table 3.

The free radical inhibition capacity (expressed as mean %inhibition) was 79% (range 69–89%) in DPPH and 29% (range 6–69%) in ABTS. The results calculated as GAE were 151 mM GAE/g d.w. (range 133–172) and 3 mM GAE/g d.w. (range 0.7–7) for DPPH and ABTS, respectively. In the DPPH, products from all regions showed an effect at a similar level of ~80% inhibition. Larger differences can be seen in the ABTS, where Turkish products showed the highest activity, 38%, compared to Greek, 26%, and Albanian, 20%. In the FRAP test, the results ranged from 110 to 337 mM Fe(II)/g d.w., with an average value of 205 mM Fe(II)/g. The highest reduction activity was observed for samples of Turkish origin with 233 mM Fe(II)/g and slightly lower for Greek with 224 mM Fe(II)/g. The lowest values were obtained for Albanian samples with 175 mM Fe(II)/g. Considering the place of origin, the differences in antioxidant activity of *C. incanus* teas revealed by ABTS and FRAP were statistically significant. In contrast, no statistically significant differences were observed with the DPPH test. Details of the antioxidant activity in the extracts can be found in Tables S12–S14 and Figure S4.

In our previous work [8], we evaluated the antioxidant activity of *C. incanus* water infusions prepared mostly from the same products used here. The infusions had more than 2.5 times lower inhibitory activity in DPPH (~25% inhibition) and slightly lower in ABTS (~27%). As with the extracts, the Turkish products showed the highest inhibitory capacity. The FRAP test also showed a much lower ability of the infusions to reduce iron compounds (134 mM/g d.w.) compared to the extracts. It is noteworthy that water infusions contained more than six times fewer polyphenols than extracts (TPC 55 vs. 350 mg/g d.w.).

Viapiana et al. [20], in the FRAP assay, obtained comparable values for 80% hydromethanolic extracts (up to 169 mM Fe^2+^). Samples of Turkish and Albanian origin showed the highest antioxidant potential compared to samples from Cyprus or of unknown origin. Similarly, the DPPH test for samples from Turkey yielded the highest results (20–97 µM TE/g d.w.) in terms of Trolox equivalents [45]. It is suggested that greater antioxidant activity is strongly related to the total amount of polyphenols, which in turn is influenced by genetic and environmental factors and country of origin [20].

Analyzing the correlation between groups of *C. incanus* polyphenols and antioxidant potential, we found (1) a strong positive correlation between TFC and FRAP (~0.8) and a moderate correlation with SPP, SET (~0.7) and SF, SPA, TPC (~0.5); (2) moderate, but weaker correlations with TFC, SF, and SPP (~0.5) were observed in ABTS; (3) results with DPPH exhibited only weak correlations except for TFC, where a moderate correlation was noted (~0.6) (Table 4 and Table S15). The above observations are consistent with those previously published for *C. incanus* water infusions [8]. However, Dimcheva and Karsheva [44], for the twelve Bulgarian *C. incanus* samples, reached a slightly higher linear correlation of DPPH results with TPC values calculated at 0.667 (*R*^2^). This could be related to the different chemical compositions of Bulgarian plant material and the smaller number of samples tested.

An in-depth analysis of the composition of *C. incanus* products showed that all groups of polyphenols are involved in its antioxidant activity. However, a significant share can be attributed to ellagitannins and flavonoids, which are the main components of this plant material. In summary, among the antioxidant tests used, FRAP best characterized the potential of *C. incanus* products. The antioxidant activity of rock-rose polyphenols is mainly due to their redox properties, which allow them to act as reducing agents, hydrogen donors, or metal ions chelators [5,6,8,45].

### 3.4. In Vitro α-Glucosidase Inhibitory Activity of C. incanus Extracts and Their Constituents

A common condition in diabetes is postprandial hyperglycemia, which, along with oxidative stress, appears to be a key element in the pathophysiology of its late complications, particularly cardiovascular disease. Therefore, achieving proper glycemic control plays a crucial role in the effective treatment of diabetes and limiting its complications [48,49]. As postprandial hyperglycemia can occur even when glycated hemoglobin (HbA1c) levels are within the standard range, it is emerging to be an important target in diabetes treatment [50]. In addition, plenty of plant-derived compounds and extracts have been found over the past few years to inhibit the activity of digestive enzymes to a considerable extent, with particular regard to α-glucosidase [51,52,53,54].

α-Glucosidase, a digestive enzyme located in the brush border of enterocytes, is capable of hydrolyzing glycosidic bonds in dietary polysaccharides to liberate glucose, subsequent intestinal absorption of which leads to a postprandial increase of blood glucose level. Consequently, its inhibitors would delay the digestion of carbohydrates and reduce the postprandial spike in blood glucose levels along with insulin secretion [55]. Therefore, owing to the utmost importance of glucose level control, the identification of substances able to target α-glucosidase offers a great strategy for treating diabetes. Currently, there are three drugs available that belong to the synthetic inhibitors, i.e., acarbose, voglibose, and miglitol, used in diabetes treatment since the 1990s [56].

In the present study, extracts of *C. incanus* teas have been examined to assess their ability to inhibit α-glucosidase. Basically, all of the tested extracts in a concentration of 250 µg/mL (drug-extract ratio μg:mL) and under the assay conditions were characterized by an exceptional ability to inhibit α-glucosidase, with the mean value reaching 97.8 ± 2.7%, and mean IC_50_ equal to 128 µg/mL. Further analysis of the selected five extracts (Ci17, Ci43, Ci48, Ci52, Ci53) showed that the inhibition presented by 1 μg/mL extract dilutions exceeded 90%, with the exception of the sample Ci52, whose inhibitory activity was noticeably lower. However, after further diluting extracts to a final concentration of 0.5 μg/mL, the lowest inhibition was exhibited by Ci48, and the highest was observed in the case of Ci43. As shown in Figure S1, inhibition of α-glucosidase by the *C. incanus* extracts occurred in a concentration-dependent manner.

**Figure 1:**
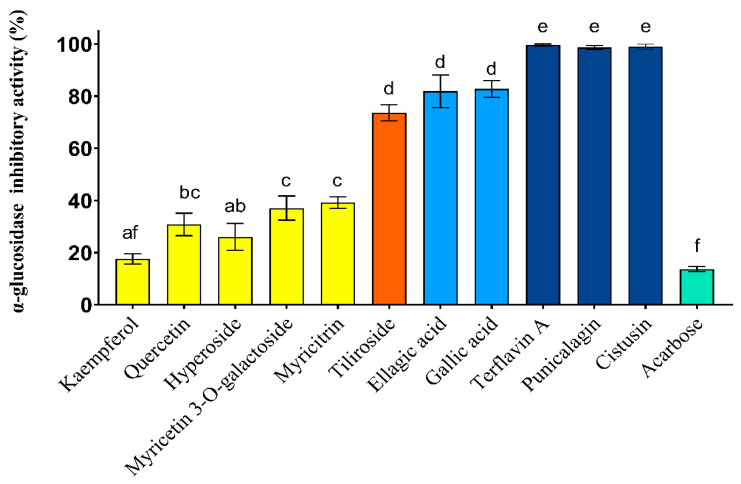
Inhibitory effect on α-glucosidase activity of *C. incanus* polyphenols. The graph shows %inhibition of compound solutions in a concentration equal to 500 µg/mL performed in triplicate ± SD. Values not sharing a common letter are significantly different at *p* < 0.05 by Tukey’s multiple comparisons tests.

The ability of each pure compound at 500 µg/mL to inhibit α-glucosidase varied (Figure 1). The tested set of compounds contained flavonol aglycones (kaempferol, quercetin, myricetin), flavonol glycosides (myricitrin, myricetin-3-*O*-galactoside, hyperoside), phenolic acids (ellagic and gallic), and ellagitannins (cistusin, punicalagin, terflavin A). Among the compounds assayed, ellagitannins exhibited the highest activity towards α-glucosidase with nearly 100% inhibition. A considerable inhibitory effect could also be seen in the case of phenolic acids (81.9% and 82.8% for ellagic and gallic acid, respectively) and tiliroside (73.6%). Moderate values (30–40% inhibition) were calculated for myricitrin, myricetin-3-*O*-galactoside, and quercetin. On the other hand, kaempferol and hyperoside presented weaker inhibitory activity (17.6% and 26% inhibition). Remarkably, all tested polyphenols exhibited stronger anti-glucosidase activity than acarbose assayed in the same concentration (13.7%). Unfortunately, myricetin could not be tested here due to its darkening under test conditions to an intense brown color (most likely due to decomposition), which limited the use of the method.

From our results, it can be noted that the ability to inhibit α-glucosidase by flavonol aglycones increases with the increasing number of hydroxyl groups. Likewise, the same trend can be observed in the case of flavonol glycosides, as hyperoside possesses fewer OH groups than myricitrin and presents significantly lower inhibitory action. The type of sugar forming the glycoside, on the other hand, does not seem to be important (myricitrin vs. myricetin-3-*O*-galactoside). Meanwhile, the esterification of glycosides was concurrent with the increase of anti-glucosidase activity, as seen in the example of tiliroside, whose effect significantly exceeds the inhibitory properties of the tested glycosides. Also, phenolic acids, represented by gallic and ellagic acids have been determined to be weaker enzyme inhibitors than ellagitannins, which are glucose esters. In this study, three ellagitannins—cistusin, punicalagin, and terflavin A—presented the highest anti-glucosidase activity among *C. incanus* polyphenols assayed with a comparable level of inhibition.

### 3.5. Mode of α-Glucosidase Inhibition by C. incanus Polyphenols and Extracts

In the next step, we investigated the inhibitory activity of *C. incanus* constituents, followed by a kinetic study aiming to determine the type of α-glucosidase inhibition shown by the six most potent among the tested polyphenols. Some have been previously demonstrated to possess anti-glucosidase activity, e.g., gallic and ellagic acids [51,52,53]. Abdelli et al. [54], in their molecular docking study, demonstrated that gallic acid interacts via numerous H-donor bonds with key amino acid residues located in the active site of the enzyme, reducing its activity. Here, based on the results of kinetic studies, we found that gallic acid inhibits α-glucosidase with an IC_50_ value equal to 117 µg/mL in a mixed mode of inhibition, which is in agreement with a recent report by Choudhary et al. [57]. Another widely studied compound is punicalagin, the main component of pomegranate, which is attributed to anti-diabetic and health-promoting properties [58]. Extensive studies also covered the ability of punicalagin to inhibit α-glucosidase [59,60], and it has been revealed that it occurs in a competitive manner via direct binding to the enzyme. It is suspected to be related to the abundance of -OH groups in its molecule, given that their presence determines the formation of hydrogen bonds with the residues of the α-glucosidase active site and subsequent blocking activity [40]. In addition, tiliroside was previously reported as a potent α-glucosidase inhibitor [61,62].

A kinetic study was conducted using increasing concentrations of p-NPG and varying concentrations of polyphenols or without the presence of any inhibitor. In order to graphically present the kinetics of enzyme inhibition, Lineweaver–Burk plots, also known as double reciprocal plots, were created (Figure S2).

Accordingly, evaluation of inhibition kinetics revealed that patterns presented by acarbose and punicalagin are typical for competitive inhibition type, while the other compounds exhibit features of both competitive and noncompetitive inhibition manifested by a change in both the Michaelis constant (K_m_) and maximum velocity of reaction (V_max_), which is consistent with the effects of a mixed inhibitor which binds both the free enzyme and the substrate–enzyme complex with different affinities.

According to the results, the most potent inhibitor among the tested compounds was cistusin, with an IC_50_ value of 0.7 µM (nevertheless, the results presented here were obtained only using in vitro methods; thus, further experiments in appropriate in vivo models will be necessary to verify the proposed indications in clinical practice Table 5), followed by punicalagin and terflavin A (1.1 µM both). Ellagic acid was the fourth inhibitor in the series, which seems relevant because it is a degradation product of ellagitannins (11 µM). In comparison, the half-maximal inhibitory concentration of acarbose, under the assay conditions, equaled 3.3 mM. Several extracts were also subjected to a kinetic study. Their mode of α-glucosidase inhibition according to the calculated parameter change has been determined as mixed, which is comprehensible as they contain a variety of compounds presenting competitive, non-competitive, or mixed inhibition. The concentrations of ellagitannins and flavonoids in *C. incanus* extracts, determined at 530–3787 μg/mL and 242–458 μg/mL, greatly exceed the IC_50_ values (μg/mL) necessary for α-glucosidase inhibition.

According to bioavailability studies, ellagitannins (such as punicalagin and punicalin) are not absorbed into the bloodstream due to their size. Instead, after consumption of ellagitannin-rich foods, they are hydrolyzed to ellagic acid and metabolized by the gut microbiota to urolithins. Taking the above into account and considering their long-term persistence in the body, these human metabolites are currently recognized bioactive forms responsible for various health-promoting effects [8,63].

Nevertheless, the results presented here were obtained only using in vitro methods; thus, further experiments in appropriate in vivo models will be necessary to verify the proposed indications in clinical practice.

## 4. Conclusions

Our results show that *C. incanus* is a rich source of polyphenols (5.5–23%), especially ellagitannins (2.5–19%), among which punicalagin, cistusin, and terflavin A were the main representatives. Flavonoids were the second most abundant group (1.2–2.3%), with myricitrin, myricetin-3-*O*-galactoside, hyperoside, and tiliroside.

*C. incanus* teas have shown high antioxidant and α-glucosidase inhibitory potential due to the presence of ellagitannins, flavonoids, and phenolic acids. The most powerful inhibitor was cistusin. Ellagic acid, which is formed abundantly in the intestine by the biotransformation of ellagitannins, also proved to be an important inhibitor. The activity of the extracts against α-glucosidase was significantly superior to acarbose regardless of differences in chemical composition and site of origin (mean IC_50_ 128 µg of plant material per 1 mL of extract). Among the products tested, those of Turkish origin exhibited the highest antioxidant potential and ellagitannin content.

Therefore, the ability to reduce oxidative stress and inhibit polysaccharide metabolism possibly determines the *C. incanus* hypoglycemic and antidiabetic effects. These properties may also reduce cardiometabolic risk. However, further in vivo studies are needed.

## Figures and Tables

**Table 1:** Contents of polyphenols, flavonoids, ellagitannins, and phenolic acids by HPLC-DAD, and total phenolic and flavonoid content by spectrophotometric methods in *C. incanus* teas.

Origin		HPLC-DAD Method				Spectrophotometric Method	
		SPP Sum of Polyphenols	SF Sum of Flavonoids	SET Sum of Ellagitannins	SPA Sum of Phenolic Acids	TPC	TFC
		[mg/g d.w.]	[mg/g d.w.]	[mg/g d.w.]	[mg/g d.w.]	[mg GAE/g d.w.]	[mg ME/g d.w.]
All (*n* = 52)	Mean	101.65	17.68	73.24	10.73	349.59	39.71
	SD	41.07	2.28	39.06	2.83	137.26	12.05
	Median	90.43	18.12	65.43	10.44	372.95	36.79
	Max	228.75	22.89	189.36	20.92	623.40	72.12
	Min	54.35	12.13	26.48	4.68	13.62	21.45
Turkey (*n* = 23)	Mean	123.71	18.42	94.10	11.18	354.80	44.68
	SD	47.53	1.96	45.40	3.20	156.50	13.48
	Median	105.47	18.62	76.22	11.15	374.13	44.67
	Max	228.75	22.89	189.36	20.92	609.58	72.12
	Min	55.35	14.54	27.79	4.68	13.62	23.20
Albania (*n* = 10)	Mean	80.18	17.33	51.82	11.03	327.70	33.47
	SD	21.27	2.52	18.86	2.18	115.45	6.13
	Median	78.60	17.82	50.07	10.54	358.91	34.06
	Max	124.68	21.13	92.78	15.82	472.57	42.12
	Min	54.76	12.64	29.17	8.67	94.36	22.75
Greece (*n* = 3)	Mean	92.27	17.85	64.14	10.27	403.43	40.38
	SD	28.00	0.70	27.28	1.38	50.76	7.34
	Median	83.23	17.79	55.53	9.90	374.92	40.56
	Max	123.67	18.58	94.69	11.80	462.03	47.63
	Min	69.90	17.19	42.21	9.11	373.34	32.95

TPC—total phenolic content; TFC—total flavonoid content; mg GAE—gallic acid equivalents; mg ME—myricetin equivalents; SD—standard deviation; Min—minimum value; Max—maximum value; g d.w.—a gram of dry weight.

**Table 2:** Content of individual polyphenolic compounds in *C. incanus* teas.

Origin		HHDP-Glc ^1,2^	Punicalin ^1,2^	Gallic Acid	Punicalagin ^1^	Terflavin A ^1^	Cistusin ^1^	Catechin	Epicatechin	Myricetin-3-*O*- galactoside	Myricetin-3-*O*- glucoside ^3^	Ellagic Acid	Myricetin-3-*O*- arabinoside ^3^	Myricitrin	Hyperoside	Isoquercitrin	Quercetin-3-*O*- arabinoside ^4^	Quercitrin	Kaempferol-3-*O*- glucoside	Helichrysoside ^1,5^	Tiliroside ^1^	Coumaroyl- tiliroside ^1,5^
		[mg/g d.w]																				
All (*n* = 52)	Mean	4.62	0.41	7.29	29.41	14.01	24.79	1.14	1.11	2.66	0.21	3.44	0.63	3.80	3.22	0.30	0.27	0.11	0.09	0.40	2.42	1.32
	SD	2.36	0.33	2.12	18.14	4.98	15.70	0.48	0.43	0.95	0.33	1.00	0.32	1.83	0.64	0.27	0.30	0.20	0.13	0.08	0.76	0.56
	Median	4.30	0.39	7.10	25.77	13.28	20.67	1.15	1.09	2.74	0.04	3.36	0.60	3.53	3.04	0.22	0.22	LOQ	0.02	0.39	2.24	1.16
	Max	13.05	1.06	16.11	82.86	33.08	74.93	2.27	2.44	4.54	1.26	5.59	1.32	8.59	5.07	0.98	1.10	0.97	0.46	0.65	4.38	2.72
	Min	0.68	LOQ	3.53	7.77	6.51	5.55	0.42	0.42	LOQ	LOQ	1.15	LOQ	0.39	2.46	LOQ	LOQ	LOQ	LOQ	0.21	1.25	0.55
Turkey (*n* = 23)	Mean	6.05	0.58	7.45	37.96	16.25	33.27	1.13	1.30	2.84	0.28	3.73	0.65	3.48	3.39	0.33	0.32	0.12	0.12	0.38	2.58	1.49
	SD	2.75	0.30	2.50	21.72	5.98	17.71	0.56	0.43	0.91	0.34	1.12	0.32	1.91	0.70	0.31	0.32	0.25	0.16	0.08	0.85	0.55
	Median	6.22	0.49	7.17	29.00	15.45	26.00	1.17	1.35	2.91	0.13	3.54	0.61	3.38	3.22	0.25	0.28	LOQ	0.03	0.38	2.54	1.52
	Max	13.05	1.06	16.11	82.86	33.08	74.93	2.27	2.44	4.16	1.26	5.52	1.28	8.59	5.07	0.98	1.10	0.97	0.46	0.61	4.38	2.48
	Min	1.97	LOQ	3.53	8.46	7.13	8.93	0.42	0.42	0.49	LOQ	1.15	0.02	0.39	2.47	LOQ	LOQ	LOQ	LOQ	0.21	1.25	0.55
Albania (*n* = 10)	Mean	3.14	0.17	7.76	19.95	13.08	15.49	1.30	0.74	2.83	0.17	3.26	0.71	4.34	3.04	0.20	0.23	0.13	0.02	0.38	2.21	1.02
	SD	1.20	0.20	1.50	8.04	3.55	7.64	0.38	0.21	0.68	0.37	0.75	0.37	1.28	0.41	0.16	0.25	0.13	0.04	0.05	0.69	0.55
	Median	3.32	0.08	7.54	19.56	12.78	15.87	1.27	0.71	2.81	LOQ	2.93	0.71	4.85	3.13	0.24	0.17	0.08	LOQ	0.38	2.11	0.88
	Max	4.52	0.59	10.86	33.05	20.39	34.24	1.81	1.08	3.92	1.03	4.96	1.28	5.80	3.82	0.45	0.69	0.34	0.14	0.45	3.99	2.54
	Min	0.68	LOQ	5.89	8.87	8.09	6.64	0.75	0.47	1.66	LOQ	2.53	LOQ	1.63	2.47	LOQ	LOQ	LOQ	LOQ	0.31	1.36	0.58
Greece (*n* = 3)	Mean	4.39	0.21	7.25	26.46	13.34	19.74	1.18	1.19	2.61	0.39	3.02	0.64	3.82	3.15	0.38	0.36	0.03	LOQ	0.33	2.55	1.23
	SD	0.17	0.20	0.77	17.16	4.39	9.84	0.44	0.35	0.62	0.67	0.72	0.21	1.04	0.62	0.26	0.36	0.04	LOQ	0.07	0.82	0.59
	Median	4.45	0.14	7.37	18.79	12.84	14.20	1.25	1.01	2.67	LOQ	2.69	0.70	4.30	3.01	0.26	0.35	LOQ	LOQ	0.36	2.13	0.97
	Max	4.53	0.43	7.95	46.11	17.96	31.10	1.58	1.60	3.19	1.16	3.85	0.82	4.54	3.83	0.67	0.73	0.08	LOQ	0.38	3.49	1.91
	Min	4.20	0.05	6.42	14.47	9.23	13.92	0.71	0.97	1.96	LOQ	2.53	0.41	2.62	2.61	0.21	LOQ	LOQ	LOQ	0.25	2.02	0.81

SD—standard deviation; HHDP-Glc—hexahydrodiphenoylglucose; Min—minimum value; Max—maximum value; g d.w.—grams of dry weight; LOQ—below the limit of quantification; ^1^ the values quoted are the sum of the isomers; ^2^ calculated as punicalagin equivalents; ^3^ calculated as myricetin-3-*O*-galactoside equivalents; ^4^ calculated as hyperoside equivalents; ^5^ calculated as tiliroside equivalents.

**Table 3:** Antioxidant activity of *C. incanus* teas.

Origin		DPPH		ABTS		FRAP	
		Inhibition	GAE	Inhibition	GAE	Fe(II)	GAE
		[%]	[mM/g d.w.]	[%]	[mM/g d.w.]	[mM/g d.w.]	[mM/g d.w.]
All (*n* = 52)	Mean	78.55 ± 5.22	151.30 ± 10.06	29.34 ± 16.70	3.17 ± 1.80	204.84 ± 51.65	45.41 ± 11.45
	Median	79.38	152.90	25.63	2.77	205.15	45.47
	Min	68.95	132.82	6.21	0.67	109.67	24.31
	Max	89.08	171.59	68.70	7.41	336.75	74.64
Turkey (*n* = 23)	Mean	79.73 ± 4.69	153.57 ± 9.59	38.22 ± 18.50	4.12 ± 2.00	230.63 ± 49.30	51.12 ± 10.93
	Median	80.96	155.95	36.67	3.96	225.46	49.98
	Min	69.34	133.56	10.28	1.11	133.27	29.54
	Max	85.93	165.52	68.70	7.41	336.75	74.64
Albania (*n* = 10)	Mean	78.95 ± 4.95	152.08 ± 9.54	19.55 ± 10.37	2.11 ± 1.17	175.22 ± 41.29	38.84 ± 9.15
	Median	80.54	155.13	18.00	1.94	168.47	37.34
	Min	69.80	134.45	6.21	0.67	109.67	24.31
	Max	86.70	167.00	40.81	4.40	234.12	51.89
Greece (*n* = 3)	Mean	78.56 ± 5.93	151.32 ± 11.42	26.01 ± 10.37	2.81 ± 1.12	223.50 ± 39.89	49.54 ± 8.84
	Median	81.85	157.65	29.28	3.16	205.29	45.50
	Min	71.71	138.13	14.40	1.55	195.97	43.44
	Max	82.11	158.16	34.35	3.71	269.24	59.68

GAE—gallic acid equivalents; Min—minimum value; Max—maximum value; g d.w.—a gram of dry weight.

**Table 4:** Correlation between chemical composition and antioxidant effect (as heat map).

Method	Group of Compounds	Linear Regression	Spearman’s Rank Order Correlation
		*R*	*R*
ABTS	Sum of polyphenols (SPP)	0.41	0.47
	Sum of flavonoids (SF)	0.47	0.52
	Sum of ellagitannins (SET)	0.37	0.43
	Sum of phenolic acids (SPA)	0.34	0.41
	Total phenolic content (TPC)	0.37	0.38
	Total flavonoid content (TFC)	0.47	0.57
DPPH	Sum of polifenolic (SPP)	0.37	0.34
	Sum of flavonoids (SF)	0.31	0.28
	Sum of ellagitannins (SET)	0.33	0.31
	Sum of phenolic acids (SPA)	0.47	0.39
	Total phenolic content (TPC)	0.33	0.35
	Total flavonoid content (TFC)	0.62	0.57
FRAP	Sum of polyphenols (SPP)	0.68	0.69
	Sum of flavonoids (SF)	0.46	0.52
	Sum of ellagitannins (SET)	0.66	0.65
	Sum of phenolic acids (SPA)	0.45	0.46
	Total phenolic content (TPC)	0.51	0.55
	Total flavonoid content (TFC)	0.78	0.77

*R*—correlation coefficient. The color intensity is proportional to correlation intensity.

**Table 5:** Half-maximal α-glucosidase inhibition (IC_50_) values of *C.incanus* polyphenols and acarbose (positive control).

Compound	Molar Mass [g/mol]	Inhibition Type	IC_50_ [µg/mL]	IC_50_ [µM]
Gallic acid	170.1	mixed	116.70	685.99
Ellagic acid	302.2	mixed	3.27	10.81
Cistusin	1252.8	mixed	0.83	0.66
Punicalagin	1084.7	competitive	1.23	1.14
Terflavin A	1086.7	mixed	1.19	1.10
Tiliroside	594.5	mixed	168.50	283.47
Acarbose	645.6	competitive	2.16 ^1^	3.34 ^2^

^1^ mg/mL; ^2^ mM

## Data Availability

The data is included in the article/supplementary data.

## Supplementary Materials

The following supporting information can be downloaded at: https://www.mdpi.com/article/10.3390/antiox12030553/s1, Section S1: Standardization of the *C. incanus* extraction process. Table S1: Comparison of mean peak areas (A-P) in methods I and II using Student’s *t*-test and Mann–Whitney U-test. Table S2: Comparison of methods I and II by Student’s *t*-test for dependent samples and Wilcoxon paired *t*-test. Table S3: Friedman’s ANOVA and Kendall’s concordance coefficient for 4 solvent concentrations. Table S4: Comparison of methanol-concentration-dependent peak areas (peaks A-P) in methods I and II using Student’s *t*-test and Mann–Whitney U-test. Section S2 Chemical composition of *C. incanus* teas. Table S5: Data on compounds identified by UHPLC-ESI-qTOF-MS in *C. incanus* teas. Table S6: Content of individual polyphenolic compounds in *C. incanus* teas. Table S7: Validation parameters of the HPLC-DAD method. Table S8: Content of total polyphenols, flavonoids, ellagitannins, phenolic acids by HPLC-DAD and spectrophotometric method, antioxidant activity, α-glucosidase inhibitory activity, and ratio of content of individual groups of compounds in *C. incanus* teas. Section S3: Ability of α-glucosidase inhibition by *C. incanus*. Figure S1: Inhibition of α-glucosidase by *C. incanus* extracts at different concentrations. Figure S2: Lineweaver–Burk plots—the inverse initial velocity as a function of the inverse of the substrate concentration—with p-NPG as a substrate of α-glucosidase inhibitory activity of tiliroside, ellagic acid, gallic acid, terflavin A, punicalagin, and cistusin. Section S4: Statistical analysis. Table S9: Test of normality of distribution. Table S10: Statistical significance between the results of the mean contents of groups of components. Figure S3: Groups of *C. incanus* components with statistically significant difference in contents. Table S11: Fisher’s LSD test for selected groups of components between countries. Table S12: Normality of distribution for antioxidant assays. Table S13: Analysis of variance (ANOVA) for antioxidant assays. Figure S4: Antioxidant tests showing statistically significant differences. Table S14: Fisher’s LSD test for ABTS and FRAP tests between countries. Table S15: Linear regression and Spearman’s rank correlation coefficient between component groups and antioxidant tests ABTS, DPPH, FRAP. Table S16: Linear regression between sum of polyphenols (SPP) and sum of ellagitannins (SET) in products of different origin. Figure S5: Correlation between sum of polyphenols (SPP) and sum of ellagitannins (SET) in products of different origin.
