# Supplementary material for: Polyphenol Profile of Cistus × incanus L. and Its Relevance to Antioxidant Effect and α-Glucosidase Inhibition
Source: Antioxidants (Basel). 2023 Feb 22;12(3):553. doi: 10.3390/antiox12030553 (PMC10045904; doi:10.3390/antiox12030553)
Supplement: Supplementary file 1 [file antioxidants-12-00553-s001.zip › antioxidants-2217028-supplementary.pdf]

antioxidants

Supplementary Materials to Article

Polyphenol profile of *Cistus × incanus* L. and its relevance to antioxidant effect and α-glucosidase inhibition

Aneta Starzec [1], Maciej Włodarczyk [1], Dominika Kunachowicz [2], Andrzej Dryś [3], Marta Kepinska [2], and Izabela Fecka[1,4,*]

[1] Department of Pharmacognosy and Herbal Medicines, Faculty of Pharmacy, Wroclaw Medical University, Borowska 211a, 50-556 Wrocław, Poland
[2] Department of Pharmaceutical Biochemistry, Division of Biomedical and Environmental Sciences, Faculty of Pharmacy, Wroclaw Medical University, Borowska 211a, 50-556 Wrocław, Poland
[3] Department of Physical Chemistry and Biophysics, Faculty of Pharmacy, Wroclaw Medical University, Borowska 211a, 50-556 Wrocław, Poland
[4] Committee for Therapeutics and Drug Sciences, Polish Academy of Sciences, Defilad 1, 00-901 Warszawa, Poland
* Correspondence: izabela.fecka@umw.edu.pl

S1. Standardization of the *C. incanus* extraction method

Optimization of the extraction process of *C. incanus* teas was carried out prior to testing the chemical composition of the flavonoid, ellagitannin and phenolic acid content. In order to choose the best way to extract polyphenolic compounds from plant material, tests were carried out for two different methods and four extraction mixtures. The first (method I) was based on extracting the compounds in a water bath, under a reflux condenser for 15 min, after reaching the boiling point. In method II, extraction was conducted in an ultrasonic bath for 15 min at temp. up to 40°C. First, for each method, 3 different concentrations of extractant (water-methanol) were compared: 50%, 60% and 70% (v/v). For each extract, approx. 1 g (to three decimal places) of the selected product Ci7 was weighed on analytical scale (Ohaus ANALYTICAL Plus, model AP110E). The prepared hydromethanolic extracts were filtered by Durapore 0.45 μm filters (Millipore). Each type of extract was performed in 6 replicates.

After a preliminary analysis of the methods, a preference was given to method I, so an additional extractant concentration of 55% was added because of the similar results for 50% and 60% aq. methanol. Subsequently, the extracts were subjected to chromatographic analysis using a Dionex Ultimate 3000 liquid chromatograph (HPLC-DAD). The obtained chromatographic data were statistically analyzed using Statistica software (StatSoft, TIBCO Software Inc.). Calculations were performed for the areas of 16 selected peaks. Methods I and II were compared in order to select the one with higher values for the majority of peaks. In addition, it was indicated which of the 4 selected solvent concentrations is the most optimal, i.e. the one in which most peaks show the highest values.

The extraction efficiency was evaluated on the basis of peak areas for peaks A-P, later identified as: A and I—gallic and ellagic acid, respectively; peaks B and F—punicalagin isomers; peaks C and G—terflavin A isomers; peaks E and H—cistusin isomers; peaks J-P—myricetin-3-*O*-galactoside, myricitrin, hyperoside, quercetin-3-*O*-arabinoside, quercitrin and tiliroside isomers, respectively. Statistical analysis by Student's t-test and Mann-Whitney U-test showed that method I yielded higher results for most peaks (A, C-E, H-P) than method II. The differences in results were statistically significant.

Table S1 shows mean peak areas in methods I and II, standard deviations and p-value. A positive test result indicates that the peak for method I is larger than the peak recorded by method II. Higher values for method II were obtained for the punicalagin isomers, cistusin isomers, and one of the terflavin A isomers; however, for most of these cases the difference was not statistically significant.

Table S1. Comparison of mean peak areas (A-P) in methods I and II using Student's t-test and Mann-Whitney U-test (p<0.05).

**Variable**	**Average peak area**				***p*-ratio**	
	Method I[1]	SD	Method II[1]	SD	Student's t-test	Mann-Whitney U-test
Peak A	23.6	1.4	16.3	2.0	0.000	0.000
Peak B	55.6	7.4	58.6	6.5	0.201	0.169
Peak C	19.0	0.4	17.9	1.8	0.016	0.211
Peak D	16.5	1.2	12.0	1.9	0.000	0.000
Peak E	28.2	3.3	30.8	2.8	0.015	0.022
Peak F	51.7	6.7	57.9	12.5	0.071	0.091
Peak G	23.4	2.4	25.0	3.8	0.123	0.159
Peak H	23.8	4.2	29.8	5.7	0.001	0.001
Peak I	38.5	1.4	30.5	1.0	0.000	0.000
Peak J	20.8	0.2	17.6	0.9	0.000	0.000
Peak K	78.5	0.6	68.1	3.2	0.000	0.000
Peak L	10.9	0.1	10.0	0.4	0.000	0.000
Peak M	7.2	0.1	6.5	0.3	0.000	0.000
Peak N	18.1	0.2	15.4	1.1	0.000	0.000
Peak O	22.5	0.5	8.4	0.6	0.000	0.000
Peak P	5.4	0.1	2.2	0.2	0.000	0.000

[1] the average from 50%, 60% and 70% aq. methanol for each peak; SD, standard deviation.

Student's t-test for dependent samples (taking into account methanol concentration) also indicates method I as more optimal. The highest peaks are observed at 50% aq. methanol however, the comparison at 70% aq. methanol is the most reliable ($p<0.05$). Similar results are obtained using the Wilcoxon paired t-test for dependent samples. The results are given in Table S2.

Table S2. Comparison of methods I and II by Student's t-test for dependent samples and Wilcoxon paired t-test ($p<0.05$).

**Variable**	**Concentration of methanol [%]**	**Average peak area**	**SD**	***p*-ratio**	
				Student's t-test	Wilcoxon paired t-test
Method I	50	29.6	20.9	0.279	0.334
Method II		28.0	22.1		
Method I	60	27.9	19.5	0.279	0.196
Method II		26.0	21.2		
Method I	70	25.7	18.2	0.026	0.026
Method II		22.4	17.0		

SD, standard deviation.

After an initial evaluation, it appeared that the most optimal concentration for method I, i.e. the one for which the peak area was greater, was methanol at 50%, but the results for 60% were very similar. Therefore, additional hydromethanolic extracts were made under reflux (method I) with 55% aq. methanol as an intermediate concentration. In the 55% hydromethanolic extracts, higher results were observed for gallic acid, myricetin-3-*O*-galactoside, myricitrin, hyperoside, quercetin-3-*O*-arabinoside and quercitrin for

both the 50% and 60% hydromethanolic extracts. Larger peak areas for the 55% extracts relative to the 60% extracts were obtained for cistusin, punicalagin and terflavin A isomers, but these were lower than for the 50% extracts. In the 60% extracts, only one of the tiliroside isomers (peak O) was higher compared to the other concentrations. To determine the most optimal concentration, the mean values of all peaks for the four concentrations (50%, 55%, 60%, 70%) were compared by performing a Friedman rank ANOVA test. The highest mean value of all peaks was obtained for the 50% aq. methanol; however, the highest peaks were observed for 55% aq. methanol. The results for this test are presented in Table S3.

In light of the above results, method I with 55% aq. methanol was considered the optimal procedure for extracting *C. incanus* products. Detailed statistical data can be found in Table S4.

Table S3. Friedman's ANOVA and Kendall's concordance coefficient for 4 solvent concentrations.

Concentration of methanol [%]	Average of ranks	Sum of ranks	Mean	SD
50	2.6	41	29.6	20.9
55	3.3	52	28.9	20.3
60	2.5	40	27.9	19.5
70	1.7	27	25.7	18.2

SD, standard deviation

Table S4. Comparison of methanol-concentration-dependent peak areas (peaks A-P) in methods I and II using Student's t-test and Mann-Whitney U-test ($p<0.05$).

Variable	Concentration of methanol [%]	Average peak area						*p*-ratio	
		Method I[1]	SD	N	Method II[1]	SD	N	Student's t-test	Mann-Whitney U-test
Peak A	50	25.0	0.2	6	18.2	0.5	6	0.000	0.005
Peak B		64.1	0.4	6	64.8	0.8	6	0.070	0.066
Peak C		19.3	0.1	6	19.9	0.3	6	0.000	0.005
Peak D		16.3	0.2	6	14.3	0.7	6	0.000	0.005
Peak E		32.1	0.5	6	33.3	0.4	6	0.001	0.013
Peak F		59.6	1.0	6	69.3	1.5	6	0.000	0.005
Peak G		26.3	0.4	6	29.1	0.6	6	0.000	0.005
Peak H		29.2	0.4	6	35.9	0.5	6	0.000	0.005
Peak I		39.3	0.2	6	31.3	0.4	6	0.000	0.005
Peak J		20.8	0.2	6	18.6	0.3	6	0.000	0.005
Peak K		78.2	0.8	6	69.6	1.3	6	0.000	0.005
Peak L		10.9	0.1	6	9.5	0.2	6	0.000	0.005
Peak M		7.2	0.1	6	6.7	0.2	6	0.001	0.013
Peak N		18.0	0.2	6	16.4	0.3	6	0.000	0.005
Peak O		21.9	0.2	6	8.0	0.5	6	0.000	0.005
Peak P		5.4	0.1	6	2.2	0.1	6	0.000	0.005
Peak A	60	24.0	0.3	6	16.9	0.6	6	0.000	0.005
Peak B		55.8	0.6	6	60.7	1.1	6	0.000	0.005
Peak C		18.5	0.2	6	17.9	1.0	6	0.143	0.378
Peak D		18.0	0.1	6	11.8	0.5	6	0.000	0.005
Peak E		28.1	0.2	6	31.7	1.6	6	0.000	0.005
Peak F		51.9	0.7	6	63.2	2.4	6	0.000	0.005
Peak G		23.2	0.3	6	22.9	3.0	6	0.839	0.378

**Variable**	**Concentration of methanol [%]**	**Average peak area**						***p*-ratio**	
		Method I[1]	SD	N	Method II[1]	SD	N	Student's t-test	Mann-Whitney U-test
Peak H		22.8	0.3	6	31.0	1.5	6	0.000	0.005
Peak I		39.4	0.2	6	30.0	1.1	6	0.000	0.005
Peak J		20.7	0.1	6	17.1	0.9	6	0.000	0.005
Peak K		78.9	0.5	6	70.7	1.0	6	0.000	0.005
Peak L		11.0	0.0	6	10.2	0.2	6	0.000	0.005
Peak M		7.2	0.0	6	6.5	0.2	6	0.000	0.005
Peak N		18.1	0.1	6	15.1	1.4	6	0.000	0.005
Peak O		23.1	0.2	6	8.4	0.1	6	0.000	0.005
Peak P		5.5	0.1	6	2.1	0.0	6	0.000	0.005
Peak A		21.8	0.6	6	13.8	0.4	6	0.000	0.005
Peak B		46.8	1.7	6	50.2	1.8	6	0.007	0.031
Peak C		19.3	0.3	6	15.9	0.7	6	0.000	0.005
Peak D		15.2	0.2	6	9.9	0.3	6	0.000	0.005
Peak E		24.4	1.1	6	27.4	0.7	6	0.000	0.005
Peak F		43.7	1.1	6	41.3	0.5	6	0.001	0.005
Peak G		20.6	0.5	6	23.1	3.1	6	0.077	0.013
Peak H	70	19.5	0.8	6	22.6	0.5	6	0.000	0.005
Peak I		36.6	0.7	6	30.3	0.8	6	0.000	0.005
Peak J		20.8	0.3	6	17.2	0.3	6	0.000	0.005
Peak K		78.5	0.4	6	64.0	0.9	6	0.000	0.005
Peak L		10.9	0.1	6	10.4	0.1	6	0.000	0.005
Peak M		7.3	0.1	6	6.2	0.1	6	0.000	0.005
Peak N		18.2	0.1	6	14.6	0.3	6	0.000	0.005
Peak O		22.6	0.3	6	8.7	0.8	6	0.000	0.005
Peak P		5.4	0.2	6	2.3	0.2	6	0.000	0.005

[1] average of 6 measurements for each peak; SD, standard deviation; N, number of samples

S2. Chemical composition of *C. incanus* teas

Table S5. Data on compounds identified by UHPLC-ESI-qTOF-MS in *C. incanus* teas.

№	Rt1 [min]	Rt2 [min]	compound identification / tentative identification	UV_{max} [nm]	$[M–H]^-$ $[M–2H]^{-2}$	∣err.∣ [ppm]	[M] formula	MS/MS fragments (collision energy)	Identification method
1	1.13	1.16	HHDP-Glc (isomer a)	260, 380	481.0627	0.6	C20 H18 O14	(38eV) 301, 275, 257	t: HRMS+MS/MS+UV
2	1.13	1.16	punicalin / GEG-Glc (isomer a)	260, 380	781.0531	0.1	C34 H22 O22	(47eV) 721, 601, 575, 449, 299, 275	nat. std., HRMS+MS/MS+UV
3	1.40	1.41	HHDP-Glc (isomer b)	260, 380	481.0621	0.6	C20 H18 O14	(38eV) 301, 275, 257	t: HRMS+MS/MS+UV
4	1.57	1.54	punicalin / GEG-Glc (isomer b)	260, 380	781.0532	0.2	C34 H22 O22	(47eV) 721, 601, 575, 449, 299, 275	nat. std., HRMS+MS/MS+UV
5	1.70	1.71	gallic acid	262	169.0142	0.3	C7 H6 O5	(30eV) 125	std.
6	7.96	6.27	punicalagin (isomer a)	258, 380	541.0239	3.9	C48 H28 O30	(32eV) 781, 763, 601, 575, 451, 425, 301, 275	isol. std. (NMR, HRMS, MS/MS)
7	8.17	6.24	terflavin A (isomer a)	overl.	542.0270	overl.	C48 H30 O30	overl.	isol. std. (NMR, HRMS, MS/MS)
8	8.47	6.73	gallocatechin	overl.	305.0661	2.0	C15 H14 O7	overl.	t: HRMS
9	8.72	7.05	cistusin (isomer a)	258, 380	625.0268	3.4	C55 H30 O25	-	isol. std. (NMR, HRMS, MS/MS)
10	8.89	7.29	punicalagin (isomer b)	258, 380	541.0245	2.7	C48 H28 O30	(32eV) 781, 763, 601, 575, 451, 425, 301, 275	isol. std. (NMR, HRMS, MS/MS)
11	8.89	7.35	catechin (CT)	overl.	289.0705	4.6	C15 H14 O6	overl.	std.
12	9.19	7.72	terflavin A (isomer b)	258, 380	542.0304	6.4	C48 H30 O30	(34eV) 781, 763, 601, 575, 451, 425, 301, 275	isol. std. (NMR, HRMS, MS/MS)
13	9.48	8.31	cistusin (isomer b)	258, 380	625.0269	3.2	C55 H30 O25	(34eV) 781, 763, 601, 575, 541($^{-2}$), 301, 275, 181	isol. std. (NMR, HRMS, MS/MS)
14	9.93	8.93	epicatechin (EC)	overl.	289.0701	5.7	C15 H14 O6	overl.	std.
15	10.12	9.77	ellagic acid hexoside	overl.	463.0527	1.8	C20 H16 O13	(37eV) 301	t: HRMS+MS/MS
16	10.32	9.77	gallocatechin gallate	258	457.0765	2.5	C22 H18 O11	(37eV) 305, 219, 169, 125	t: HRMS+MS/MS+UV
17	10.50	10.63	ellagic acid pentoside (isomer a)	overl.	433.0421	1.9	C19 H14 O12	overl.	t: HRMS
18	10.50	10.56	myricetin-hexoside gallate	overl.	631.0953	1.2	C28 H24 O17	(42eV) 479, 316	t: HRMS+MS/MS
19	10.59	10.63	methylated flavogallic acid	256	483.0211	0.9	C22 H12 O13	(38eV) 451	t: HRMS+MS/MS
20	10.85	11.34	myricetin-3-*O*-galactoside (gmelinoside I)	260, 356	479.0821	2.1	C21 H20 O13	(38eV) 316>315	isol. std. (NMR, HRMS, MS/MS)
21	10.89	11.56	myricetin-3-*O*-glucoside (isomyricitrin)	260, 356	479.0815	3.4	C21 H20 O13	(38eV) 316>315	nat. std., HRMS+MS/MS+UV
22	11.01	12.05	ellagic acid pentoside (isomer b)	overl.	433.0421	1.9	C19 H14 O12	(37eV) 301	t: HRMS+MS/MS
23	11.05	11.66	CAS: 1891055-64-3	258, 275	493.1349	0.5	C23 H26 O12	(38eV) 313, 301, 179, 169	literature: HRMS+MS/MS+UV
24	11.21	12.59	ellagic acid pentoside (isomer c)	overl.	433.0427	3.2	C19 H14 O12	(37eV) 301	t: HRMS+MS/MS
25	11.24	12.68	ellagic acid deoxyhexoside	overl.	447.0582	3.0	C20 H16 O12	(37eV) 301	t: HRMS+MS/MS
26	11.28	12.50	quercetin-3-*O*-hexoside gallate	256	615.1008	2.6	C28 H24 O16	(42eV) 463, 300, 169	t: HRMS+MS/MS+UV
27	11.43	12.81	myricetin-3-*O*-arabinopyranoside (—)	266, 360	449.0718	1.8	C20 H18 O12	(37eV) 316>315	isol. std. (NMR, HRMS, MS/MS)
28	11.44	13.20	quercetin-3-*O*-2″-rhamnoglucoside (rutin)	overl.	609.1348	3.8	C27 H30 O16	overl.	std.
29	11.51	12.87	myricetin-3-*O*-arabinofuranoside (betmidin)	overl.	449.0755	6.6	C20 H18 O12	(37eV) 316>315	nat. std., HRMS+MS/MS

№	Rt1 [min]	Rt2 [min]	compound identification / tentative identification	UV_{max} [nm]	$[M–H]^–$ <u>$[M–2H]^{–2}$</u>	ǀerr.ǀ [ppm]	[M] formula	MS/MS fragments (collision energy)	Identification method
30	11.56	13.14	myricetin-3-*O*-rhamnoside (myricitrin)	254, 356	463.0872	2.1	C21 H20 O12	(37eV) 316>315	isol. std. (NMR, HRMS, MS/MS)
31	11.59	13.03	ellagic acid	overl.	300.9985	1.5	C14 H6 O8	overl.	std.
32	11.66	13.49	quercetin-3-*O*-galactoside (hyperoside)	256, 356	463.0877	1.0	C21 H20 O12	(37eV) 300>301	isol. std. (NMR, HRMS, MS/MS)
33	11.75	13.81	quercetin-3-*O*-glucoside (isoquercitrin)	256, 356	463.0872	2.1	C21 H20 O12	(37eV) 300>301	std.
34	12.10	14.62	quercetin-3-*O*-xyloside (guajiverin)	-	433.0770	1.5	C20 H18 O11	-	nat. std., HRMS+MS/MS
35	12.19	14.99	kaempferol-3-*O*-galactoside (trifolin)	256, 356	447.0921	2.7	C21 H20 O11	(37eV) 284>285	nat. std., HRMS+MS/MS+UV
36	12.24	15.03	quercetin-3-*O*-arabinopyranoside (—)	256, 356	433.0764	2.9	C20 H18 O11	(37eV) 300>301	literature: HRMS+MS/MS+UV
37	12.35	15.36	quercetin-3-*O*-arabinofuranoside (avicularin)	356	433.0762	3.3	C20 H18 O11	(37eV) 300>301	nat. std., HRMS+MS/MS+UV
38	12.41	14.84	unidentified (1)	-	289.1109	2.2	C13 H22 O5 S	-	HRMS
39	12.42	15.71	kaempferol-3-*O*-glucoside (astragalin)	-	447.0917	3.5	C21 H20 O11	(37eV) 284>285	std.
40	12.52	15.87	quercetin-3-*O*-rhamnoside (quercitrin)	256, 356	447.0923	2.2	C21 H20 O11	(37eV) 300>301	std.
41	12.62	15.93	unidentified (2), sulfonate	-	439.1043	5.4	C20 H24 O9 S	(37eV) 439, 359, 314	HRMS+MS/MS
42	12.68	14.99	unidentified (3)	-	187.0972	1.8	C9 H16 O4	(30eV) 125	HRMS+MS/MS
43	12.78	16.56	K-3-*O*-pentoside	-	417.0840	3.1	C20 H18 O10	(37eV) 284	nat. std., HRMS+MS/MS
44	12.95	16.94	catechin/epicatechin-deoxyhexoside	-	435.1266	7.2	C21 H24 O10	-	t: HRMS
45	13.80	19.25	unidentified (4)	-	419.0973	2.1	C20 H20 O10	(36eV) 152	HRMS+MS/MS
46	13.92	20.35	helichrysoside (isomer a)	316	609.1241	1.4	C30 H26 O14	(42eV) 463, 300>301, 163, 145	nat. std., HRMS+MS/MS+UV
47	14.08	20.78	helichrysoside (isomer b, main)	316	609.1229	3.5	C30 H26 O14	(42eV) 463, 300>301, 163, 145	t: HRMS+MS/MS+UV
48	14.42	21.71	helichrysoside (isomer c)	316	609.1245	0.7	C30 H26 O14	-	t: HRMS+UV
49	14.58	22.18	tiliroside (isomer a) or buddlenoid A (7-*O*-)	-	593.1275	4.3	C30 H26 O13	(42eV) 447, 284>285, 163, 145	t: HRMS+MS/MS+UV
50	14.69	22.63	tiliroside (isomer b, main)	266, 316	593.1284	2.7	C30 H26 O13	(42eV) 447, 285>284, 163, 145	isol. std. (NMR, HRMS, MS/MS)
51	14.94	23.28	tiliroside (isomer c)	316	593.1287	2.3	C30 H26 O13	(42eV) 447, 285>284, 163, 145	isol. std. (NMR, HRMS, MS/MS)
52	18.41	25.34	coumaroyl-tiliroside (isomer a)	316	739.1650	2.5	C39 H32 O15	(45eV) 593, 575, 285>284, 163, 145	t: HRMS+MS/MS+UV
53	18.59	25.34	coumaroyl-tiliroside (isomer b, main)	316	739.1653	2.0	C39 H32 O15	(45eV) 593, 575, 285>284, 163, 145	t: HRMS+MS/MS+UV
54	18.76	25.34	coumaroyl-tiliroside (isomer c)	-	739.1651	2.3	C39 H32 O15	-	t: HRMS

Rt1 and Rt2 refer to different UHPLC-MS retention times of compounds for different gradients [31] and [18], respectively. Abbreviations: $[M−H]^–$, monodeprotonated molecule ion; $[M−2H]^{–2}$, doubly deprotonated molecule ion (underlined values in parent ion column); [M], neutral molecule; CAS, Chemical Abstracts number; CT, catechin; EC, epicatechin; HHDP-Glc, hexahydrodiphenoylglucose; isol. std., isolated standard; nat. std., confirmed by co-chromatography with extract from a well-known plant source of this compound, details in in chapter 2.1; overl., overlayed; std., commercial standard; t, tentatively identified.

Table S6. Content of individual polyphenolic compounds in *C. incanus* teas.

Origin	Extract number	HHDP-Glc [1,2]	Punicalin [1,2]	Gallic acid	Punicalagin [1]	Terflavin A [1]	Cistusin [1]	Catechin	Epicatechin	Myricetin-3-*O*-galactoside	Myricetin-3-*O*-glucoside [3]	Ellagic acid	Myricetin-3-*O*-arabinoside [3]	Myricitrin	Hyperoside	Isoquercitrin	Quercetin-3-*O*-arabinoside [4]	Quercitrin	Kaempferol-3-*O*-glucoside	Helichrysoside [1,5]	Tiliroside [1]	Coumaroyl-tiliroside [1,5]
		[mg/g d.w]																				
A	Ci1	3.29	0.02	5.89	8.87	10.34	6.64	0.96	0.61	3.05	LOQ	2.83	0.61	5.01	2.64	0.26	0.05	0.09	0.14	0.36	2.10	0.99
T	Ci2	5.84	0.49	8.45	25.60	15.35	24.29	1.49	1.40	3.26	0.10	3.43	0.50	4.21	3.18	0.27	0.30	0.05	LOQ	0.38	2.63	1.55
T	Ci3	11.82	1.05	6.53	48.49	24.41	39.72	1.31	1.36	4.16	0.32	5.32	0.59	2.74	3.82	0.45	0.44	LOQ	0.08	0.43	2.65	1.93
T	Ci4	6.09	0.44	7.81	25.58	14.48	21.33	1.16	1.44	2.34	0.07	3.41	0.16	2.29	2.91	0.22	0.21	LOQ	0.01	0.35	3.99	2.35
T	Ci5	13.05	0.90	6.39	57.98	33.08	47.60	1.57	1.07	3.70	0.21	4.49	1.01	1.28	5.07	0.83	1.02	LOQ	0.25	0.45	1.37	0.64
T	Ci6	7.21	0.31	7.07	18.59	13.09	17.03	1.02	0.89	2.79	0.09	3.08	0.31	3.59	2.82	0.22	0.18	LOQ	0.03	0.38	3.01	1.70
N	Ci7	3.45	0.01	6.38	8.63	9.38	6.12	1.15	0.65	2.67	LOQ	2.31	0.49	8.01	2.72	0.19	0.08	0.61	LOQ	0.40	1.74	0.84
N	Ci8	2.81	1.06	6.30	7.77	9.29	5.55	1.14	0.63	2.68	LOQ	2.11	0.52	7.78	2.67	0.11	0.09	0.58	LOQ	0.41	1.91	0.94
T	Ci9	2.59	LOQ	5.26	13.41	15.01	19.47	2.27	0.42	3.74	LOQ	1.75	1.28	7.02	2.64	LOQ	0.27	0.61	LOQ	0.31	2.18	0.88
T	Ci10	5.87	0.61	9.79	29.20	16.74	31.94	1.80	1.09	3.60	LOQ	3.93	0.50	5.10	3.25	0.07	0.30	0.25	0.02	0.42	1.25	1.40
T	Ci11	6.84	0.93	7.34	44.32	20.83	40.23	1.62	1.30	3.88	LOQ	5.52	0.59	3.20	3.54	0.20	0.35	LOQ	0.02	0.42	2.35	1.49
T	Ci12	3.40	0.62	10.50	32.13	19.79	30.43	2.05	1.90	4.11	LOQ	4.59	0.86	4.23	3.40	0.17	0.42	0.03	LOQ	0.38	2.24	1.19
T	Ci13	6.34	0.42	7.67	23.00	11.53	19.35	0.83	1.17	2.09	0.11	3.18	0.02	1.88	2.76	0.28	LOQ	LOQ	0.09	0.36	3.90	2.36
T	Ci14	7.85	0.48	8.94	25.94	15.54	24.75	1.18	1.12	3.44	0.15	3.54	0.70	3.35	3.27	0.38	0.43	0.14	0.33	0.38	2.94	1.58
G	Ci15	4.53	0.05	7.37	18.79	17.96	14.20	1.58	0.97	3.19	LOQ	2.53	0.82	4.30	3.01	0.21	0.35	LOQ	LOQ	0.38	2.02	0.97
A	Ci16	4.24	LOQ	7.30	16.00	13.46	12.33	1.74	1.08	3.92	LOQ	2.64	0.92	5.80	3.27	0.30	0.52	0.34	0.04	0.40	1.96	0.84
A	Ci17	4.52	0.59	10.86	33.05	20.39	34.24	1.65	0.74	2.75	LOQ	4.96	0.60	3.12	3.18	0.33	0.35	LOQ	LOQ	0.31	2.19	0.85
A	Ci18	3.14	LOQ	9.76	27.81	17.20	17.35	1.81	0.68	3.80	LOQ	3.86	1.28	4.16	3.82	0.45	0.69	0.05	0.05	0.37	1.69	0.66
A	Ci19	1.92	0.10	7.72	10.64	10.20	9.35	1.30	0.61	2.16	LOQ	2.86	0.28	3.39	2.55	LOQ	0.02	LOQ	LOQ	0.40	1.36	0.58
N	Ci20	2.44	0.39	8.10	22.16	15.28	17.79	1.62	1.17	3.11	LOQ	3.27	0.50	3.96	2.99	0.22	0.26	0.04	0.03	0.40	2.82	1.46
N	Ci21	1.67	LOQ	6.77	10.51	10.17	8.83	1.54	0.70	2.16	LOQ	2.99	0.27	4.54	2.62	0.17	0.19	0.12	LOQ	0.38	1.51	0.67
A	Ci22	0.68	LOQ	7.61	12.94	12.61	9.23	1.61	0.47	2.97	LOQ	2.89	0.63	4.86	2.93	0.14	0.36	0.30	LOQ	0.45	2.48	1.12
A	Ci23	4.12	0.28	7.27	17.54	8.09	16.00	0.75	0.49	1.66	LOQ	2.97	LOQ	1.63	2.47	0.33	LOQ	LOQ	0.01	0.36	3.99	2.54
T	Ci24	1.97	0.30	7.36	17.48	15.62	16.75	1.46	0.73	3.15	LOQ	3.26	0.63	4.59	3.14	0.16	0.24	0.11	LOQ	0.35	1.65	0.75
N	Ci25	1.66	LOQ	7.06	10.62	12.87	9.58	1.50	0.71	2.94	LOQ	2.94	0.68	5.63	2.77	LOQ	0.18	0.28	LOQ	0.39	2.23	1.04
N	Ci26	1.93	0.09	7.90	12.95	14.30	10.46	1.33	0.70	2.99	LOQ	3.40	0.70	4.92	2.90	0.18	0.25	0.12	LOQ	0.40	2.35	1.08
N	Ci27	5.52	1.01	6.56	45.19	17.72	37.72	1.28	1.60	4.54	0.19	3.71	0.90	1.43	4.98	0.85	0.55	LOQ	0.23	0.41	1.98	1.42

Origin	Extract number	HHDP-Glc [1,2]	Punicalin [1,2]	Gallic acid	Punicalagin [1]	Terflavin A [1]	Cistusin [1]	Catechin	Epicatechin	Myricetin-3-*O*-galactoside	Myricetin-3-*O*-glucoside [3]	Ellagic acid	Myricetin-3-*O*-arabinoside [3]	Myricitrin	Hyperoside	Isoquercitrin	Quercetin-3-*O*-arabinoside [4]	Quercitrin	Kaempferol-3-*O*-glucoside	Helichrysoside [1,5]	Tiliroside [1]	Coumaroyl-tiliroside [1,5]
		[mg/g d.w]																				
N	Ci28	3.56	0.52	9.93	24.21	14.46	23.24	1.96	0.99	3.09	LOQ	4.27	0.44	2.65	3.28	0.18	0.05	LOQ	0.15	0.33	1.67	0.96
N	Ci29	6.07	0.57	12.98	32.80	14.19	20.01	1.02	0.87	4.12	LOQ	5.59	1.32	3.45	4.41	0.81	1.05	LOQ	0.30	0.36	1.81	0.70
G	Ci30	4.45	0.14	6.42	14.47	9.23	13.92	1.25	1.01	2.67	LOQ	2.69	0.41	4.54	2.61	0.26	LOQ	0.08	LOQ	0.36	3.49	1.91
T	Ci31	3.74	0.43	5.82	28.79	9.98	22.42	0.44	1.34	1.36	LOQ	3.35	0.41	3.46	2.81	0.36	LOQ	LOQ	0.14	0.41	3.61	1.99
N	Ci32	3.53	0.34	5.51	28.43	9.66	23.87	0.56	1.16	0.99	0.04	2.90	0.18	2.58	2.83	0.47	LOQ	LOQ	0.12	0.46	3.44	2.04
A	Ci33	2.23	0.06	6.14	21.59	13.77	16.45	1.05	0.78	2.73	LOQ	2.53	0.95	5.30	3.07	LOQ	LOQ	0.11	LOQ	0.45	2.07	0.81
N	Ci34	4.82	0.53	6.52	35.61	11.65	32.44	0.67	1.00	2.06	0.12	3.86	0.59	2.93	3.09	0.24	LOQ	LOQ	0.03	0.42	1.87	1.13
N	Ci35	4.21	0.39	6.28	27.51	11.10	24.06	0.76	1.25	1.74	0.19	3.36	0.53	3.80	2.85	0.33	LOQ	LOQ	0.13	0.56	2.69	1.60
T	Ci36	7.26	0.92	7.20	73.61	17.88	61.25	0.42	2.44	2.11	0.60	3.98	0.82	1.02	4.65	0.98	1.10	LOQ	0.37	0.31	1.26	0.55
T	Ci37	6.60	0.89	7.14	57.22	15.08	45.82	0.43	1.43	2.14	0.50	4.40	0.52	2.65	3.49	0.64	0.34	LOQ	0.40	0.39	2.72	1.81
A	Ci38	3.34	0.26	7.55	26.82	12.96	17.54	0.93	0.96	2.87	0.69	3.31	0.98	4.85	3.22	0.22	0.29	0.07	LOQ	0.31	2.12	0.93
N	Ci39	2.09	0.06	4.87	14.84	8.39	10.07	0.74	0.65	1.72	0.27	2.08	0.46	6.30	2.74	0.09	LOQ	0.26	LOQ	0.35	1.50	0.70
G	Ci40	4.20	0.43	7.95	46.11	12.84	31.10	0.71	1.60	1.96	1.16	3.85	0.70	2.62	3.83	0.67	0.73	LOQ	LOQ	0.25	2.13	0.81
T	Ci41	2.60	0.32	6.63	26.09	11.27	21.77	1.49	0.66	2.71	0.55	3.19	0.90	5.39	2.96	0.32	LOQ	0.39	LOQ	0.32	2.13	0.96
N	Ci42	4.80	0.42	5.16	30.50	8.92	21.46	0.56	1.25	0.95	0.19	2.62	0.31	1.58	2.97	0.62	LOQ	LOQ	0.23	0.52	2.44	1.49
A	Ci43	3.88	0.35	7.53	24.24	11.75	15.74	1.24	0.96	2.42	1.03	3.81	0.80	5.33	3.22	LOQ	LOQ	0.29	LOQ	0.43	2.15	0.90
T	Ci44	5.84	0.41	6.04	33.37	11.01	27.24	0.66	1.40	2.04	0.31	3.54	0.51	3.64	3.13	LOQ	LOQ	0.01	0.39	0.46	2.87	1.64
T	Ci45	7.41	0.89	7.49	71.80	19.13	61.04	0.51	1.50	2.92	0.82	5.37	0.91	2.77	3.93	0.76	0.41	LOQ	0.46	0.21	2.25	1.44
T	Ci46	2.71	0.30	3.53	8.46	7.39	8.93	1.42	1.16	2.53	0.41	1.15	0.97	8.59	2.73	LOQ	LOQ	0.97	LOQ	0.37	2.74	1.00
N	Ci47	5.19	LOQ	5.59	29.75	10.33	23.46	0.64	1.34	1.70	0.11	3.43	0.59	3.48	2.94	LOQ	LOQ	LOQ	0.22	0.57	3.21	1.80
T	Ci48	4.63	0.23	4.34	21.73	7.13	19.12	0.62	1.45	0.49	LOQ	2.25	0.21	1.82	2.47	LOQ	LOQ	LOQ	LOQ	0.61	4.38	2.48
T	Ci49	6.35	0.71	6.61	69.41	19.70	56.46	0.65	1.57	3.05	0.70	4.50	1.16	3.42	3.88	LOQ	0.25	LOQ	0.09	0.37	2.27	1.49
N	Ci50	4.35	0.25	4.20	19.86	6.51	16.35	0.54	1.38	LOQ	0.04	1.78	LOQ	0.40	2.46	LOQ	LOQ	LOQ	0.02	0.65	3.91	2.72
T	Ci51	7.02	1.06	16.11	82.86	23.49	74.93	0.50	1.68	2.91	1.26	4.80	0.79	0.39	4.64	0.91	0.87	LOQ	LOQ	0.40	2.46	1.68
N	Ci52	4.59	0.76	5.61	54.12	16.07	51.86	0.84	2.08	2.31	0.91	4.36	0.84	2.41	3.85	0.66	0.66	LOQ	0.29	0.43	2.23	1.48

A, Albania; G, Greece; T, Turkey; N, of unknown origin; HHDP-Glc, hexahydrodiphenoylglucose; g d.w., a gram of dry weight; LOQ, below the limit of quantification; [1] the values quoted are the sum of the isomers; [2] calculated as punicalagin equivalents; [3] calculated as myrcetin-3-*O*-galactoside equivalents; [4] calculated as hyperoside equivalents; [5] calculated as tiliroside equivalents.

Table S7. Validation parameters of the HPLC-DAD method.

Compound	λ [nm]	Linear Equation	R^2	LOD [µg/mL]	LOQ [µg/mL]
gallic acid	280	y = 182.73x + 0.2733	0.9998	1.1	3.4
ellagic acid	254	y = 668.22x + 4.4156	0.9990	8.9	26.9
cistusin	254	y = 318.16x + 6.2633	0.9996	2.7	8.0
punicalagin	254	y = 508.48x + 2.3601	0.9990	8.1	24.6
terflavin A	254	y = 226.69x + 3.553	0.9998	1.2	3.7
catechin	280	y = 204.92x - 1.0976	0.9961	5.3	16.1
epicatechin	280	y = 209.63x - 1.1295	0.9991	2.7	8.2
procyanidin A1	280	y = 199.45x - 1.3096	0.9989	2.8	8.4
procyanidin B1	280	y = 82.39x - 0.2731	0.9993	0.9	2.8
procyanidin B2	280	y = 101.73x - 0.6216	0.9983	1.7	5.1
procyanidin C1	280	y = 78.32x + 0.3785	0.9982	1.4	4.3
myricetin	254	y = 559.31x + 7.9316	0.9988	8.1	24.4
myricitrin	254	y = 648.51x + 11.286	0.9996	5.7	17.3
myricetin-3-*O*-β-galactoside	254	y = 485.37x + 10.69	0.9992	13.8	41.8
myricetin-3-*O*-β-glucuronoside	254	y = 338.35x + 7.6699	0.9995	3.1	9.4
quercetin	254	y = 1206.3x + 18.384	0.9989	17.0	51.6
hyperoside	254	y= 824.01x + 21.739	0.9995	7.4	22.4
isoquercitrin	254	y = 600.53x + 13.536	0.9998	3.4	10.4
quercitrin	254	y = 774.26x + 21.252	0.9982	13.6	41.4
quercetin-3-*O*-β-glucuronoside	254	y = 410.84x + 6.4025	0.9987	9.8	29.6
kaempferol-3-*O*-β-glucuronosid	254	y = 584.24x + 9.4489	0.9950	17.3	52.4
tiliroside	320	y = 922.08x - 1.1096	0.9991	11.3	34.1

λ, wavelength; y = ax + b; y, peak area; R^2, coefficient of determination; LOD, limit of detection; LOQ, limit of quantitation

Table S8. Content of total polyphenols, flavonoids, ellagitannins, phenolic acids by HPLC-DAD and spectrophotometric method, antioxidant activity, α-glucosidase inhibitory activity and ratio of content of individual groups of compounds in *C. incanus* teas.

		**HPLC method**				**Spectrophotometric method**		**Antioxidant activity**						**α-Glucosidase inhibitory activity**		**HPLC results / Spectrophotometric results ratios**							
		SPP	SF	SET	SPA	TPC	TFC	ABTS		DPPH		FRAP				TPC/SPP	TFC/SF	SPP/SF	SPP/SET	SET/SF	SF in SPP	SET in SPP	SPA in SPP
**Origin**	**Extract number**	[mg/g d.w]				[mg GAE/g d.w.]	[mg ME/g d.w.]	[% inhibition]	[mM GAE/g]	[% inhibition]	[mM GAE/g]	[mM Fe]	[mM GAE]	[% inhibition]	[µg/mL]						[%]	[%]	[%]
A	Ci1	54.76	16.87	29.17	8.72	94.36	30.59	40.81	4.40	86.70	167.00	145.99	32.36	98.74	126.60	1.7	1.8	3.2	1.9	1.7	30.8	53.3	15.9
T	Ci2	102.79	19.33	71.58	11.89	273.76	40.69	46.97	5.07	82.74	159.38	195.63	43.36	86.16	145.08	2.7	2.1	5.3	1.4	3.7	18.8	69.6	11.6
T	Ci3	157.64	20.30	125.49	11.85	375.71	57.62	64.89	7.00	80.13	154.35	336.75	74.64	98.45	126.97	2.4	2.8	7.8	1.3	6.2	12.9	79.6	7.5
T	Ci4	96.65	17.50	67.93	11.22	179.38	37.65	48.62	5.25	84.47	162.71	205.03	45.45	98.87	126.43	1.9	2.2	5.5	1.4	3.9	18.1	70.3	11.6
T	Ci5	181.97	18.47	152.62	10.88	540.84	59.73	62.72	6.77	80.70	155.44	296.74	65.77	95.48	130.91	3.0	3.2	9.9	1.2	8.3	10.1	83.9	6.0
T	Ci6	83.40	17.02	56.23	10.15	564.67	31.60	55.06	5.94	82.99	159.85	213.75	47.38	87.52	142.82	6.8	1.9	4.9	1.5	3.3	20.4	67.4	12.2
N	Ci7	55.83	19.55	27.59	8.69	444.51	30.98	46.16	4.98	81.74	157.45	208.45	46.20	99.31	125.87	8.0	1.6	2.9	2.0	1.4	35.0	49.4	15.6
N	Ci8	54.35	19.46	26.48	8.41	552.93	27.18	43.22	4.66	84.06	161.93	189.03	41.90	98.30	127.16	10.2	1.4	2.8	2.1	1.4	35.8	48.7	15.5
T	Ci9	79.11	21.62	50.48	7.01	302.46	31.22	54.19	5.85	76.74	147.82	227.01	50.32	99.10	126.14	3.8	1.4	3.7	1.6	2.3	27.3	63.8	8.9
T	Ci10	117.11	19.04	84.35	13.71	464.44	47.61	68.70	7.41	81.77	157.51	266.42	59.05	99.33	125.85	4.0	2.5	6.1	1.4	4.4	16.3	72.0	11.7
T	Ci11	144.97	18.96	113.15	12.85	609.58	54.61	49.79	5.37	79.92	153.93	287.55	63.74	96.39	129.69	4.2	2.9	7.6	1.3	6.0	13.1	78.1	8.9
T	Ci12	122.44	20.97	86.37	15.09	418.10	44.94	62.68	6.76	83.83	161.48	249.62	55.33	98.93	126.35	3.4	2.1	5.8	1.4	4.1	17.1	70.5	12.3
T	Ci13	87.34	15.84	60.65	10.85	269.87	31.87	42.72	4.61	73.38	141.34	183.85	40.75	99.41	125.74	3.1	2.0	5.5	1.4	3.8	18.1	69.4	12.4
T	Ci14	106.45	19.39	74.57	12.49	440.02	44.40	25.69	2.77	82.45	158.81	218.44	48.42	99.08	126.17	4.1	2.3	5.5	1.4	3.8	18.2	70.1	11.7
G	Ci15	83.23	17.79	55.53	9.90	462.03	32.95	14.40	1.55	71.71	138.13	205.29	45.50	99.37	125.79	5.6	1.9	4.7	1.5	3.1	21.4	66.7	11.9
A	Ci16	77.10	21.13	46.04	9.93	417.69	33.23	17.29	1.87	80.48	155.03	215.63	47.80	99.54	125.58	5.4	1.6	3.6	1.7	2.2	27.4	59.7	12.9
A	Ci17	124.68	16.07	92.78	15.82	408.30	42.12	18.70	2.02	80.92	155.87	209.82	46.51	98.68	126.68	3.3	2.6	7.8	1.3	5.8	12.9	74.4	12.7
A	Ci18	98.64	19.52	65.50	13.62	398.77	41.16	31.43	3.39	76.37	147.10	234.12	51.89	99.26	125.93	4.0	2.1	5.1	1.5	3.4	19.8	66.4	13.8
A	Ci19	55.44	12.64	32.22	10.58	472.57	25.84	9.51	1.03	82.85	159.58	155.64	34.50	98.42	127.00	8.5	2.0	4.4	1.7	2.5	22.8	58.1	19.1

		**HPLC method**				**Spectrophotometric method**		**Antioxidant activity**						**α-Glucosidase inhibitory activity**		**HPLC results / Spectrophotometric results ratios**							
		SPP	SF	SET	SPA	TPC	TFC	ABTS		DPPH		FRAP				TPC/SPP	TFC/SF	SPP/SF	SPP/SET	SET/SF	SF in SPP	SET in SPP	SPA in SPP
**Origin**	**Extract number**	[mg/g d.w]				[mg GAE/g d.w.]	[mg ME/g d.w.]	[% inhibition]	[mM GAE/g]	[% inhibition]	[mM GAE/g]	[mM Fe]	[mM GAE]	[% inhibition]	[µg/mL]						[%]	[%]	[%]
N	Ci20	88.00	18.58	58.05	11.37	439.80	36.91	39.15	4.22	73.27	141.14	167.84	37.20	99.20	126.01	5.0	2.0	4.7	1.5	3.1	21.1	66.0	12.9
N	Ci21	55.81	14.87	31.18	9.76	273.93	26.71	6.58	0.71	69.87	134.58	111.72	24.76	98.89	126.41	4.9	1.8	3.8	1.8	2.1	26.6	55.9	17.5
A	Ci22	64.27	18.32	35.45	10.50	319.05	31.50	9.12	0.98	72.52	139.68	135.32	29.99	96.98	128.90	5.0	1.7	3.5	1.8	1.9	28.5	55.2	16.3
A	Ci23	70.48	14.22	46.02	10.24	273.25	22.75	6.21	0.67	69.80	134.45	109.67	24.31	98.99	126.27	3.9	1.6	5.0	1.5	3.2	20.2	65.3	14.5
T	Ci24	79.71	16.97	52.12	10.63	282.66	30.97	11.25	1.21	69.34	133.56	133.27	29.54	93.93	133.08	3.5	1.8	4.7	1.5	3.1	21.3	65.4	13.3
N	Ci25	63.07	18.35	34.72	10.00	322.13	29.24	12.89	1.39	71.25	137.25	143.14	31.73	98.93	126.35	5.1	1.6	3.4	1.8	1.9	29.1	55.0	15.9
N	Ci26	68.94	17.92	39.72	11.30	343.22	28.71	10.42	1.12	70.82	136.42	122.57	27.17	99.03	126.22	5.0	1.6	3.8	1.7	2.2	26.0	57.6	16.4
N	Ci27	137.80	20.37	107.15	10.27	623.40	56.50	24.77	2.67	78.85	151.87	237.89	52.73	92.19	135.59	4.5	2.8	6.8	1.3	5.3	14.8	77.8	7.5
N	Ci28	95.95	15.76	65.99	14.20	428.57	54.49	31.95	3.45	87.37	168.29	274.14	60.76	97.69	127.96	4.5	3.5	6.1	1.5	4.2	16.4	68.8	14.8
N	Ci29	112.43	20.22	73.64	18.56	507.58	62.50	36.90	3.98	89.08	171.59	281.11	62.31	98.88	126.42	4.5	3.1	5.6	1.5	3.6	18.0	65.5	16.5
G	Ci30	69.90	18.58	42.21	9.11	373.34	40.56	29.28	3.16	81.85	157.65	269.24	59.68	98.99	126.27	5.3	2.2	3.8	1.7	2.3	26.6	60.4	13.0
T	Ci31	90.85	16.32	65.36	9.17	115.12	53.13	28.55	3.08	85.93	165.52	233.48	51.75	99.25	125.94	1.3	3.3	5.6	1.4	4.0	18.0	71.9	10.1
N	Ci32	89.11	14.88	65.83	8.41	233.92	37.06	16.67	1.80	76.39	147.13	165.11	36.60	99.06	126.18	2.6	2.5	6.0	1.4	4.4	16.7	73.9	9.4
A	Ci33	80.10	17.33	54.10	8.67	236.20	34.88	14.46	1.56	80.59	155.24	181.31	40.19	99.21	126.00	2.9	2.0	4.6	1.5	3.1	21.6	67.5	10.8
N	Ci34	109.57	14.14	85.05	10.38	253.75	41.11	20.25	2.18	74.48	143.47	171.43	38.00	98.90	126.39	2.3	2.9	7.7	1.3	6.0	12.9	77.6	9.5
N	Ci35	93.33	16.42	67.27	9.64	194.12	36.91	21.41	2.31	76.80	147.92	162.83	36.09	99.21	126.00	2.1	2.2	5.7	1.4	4.1	17.6	72.1	10.3
T	Ci36	188.75	16.63	160.94	11.18	498.03	65.06	30.42	3.28	84.99	163.71	264.45	58.62	98.20	127.29	2.6	3.9	11.3	1.2	9.7	8.8	85.3	5.9
T	Ci37	154.61	17.46	125.62	11.53	372.55	53.27	24.17	2.61	78.64	151.47	203.97	45.21	97.71	127.93	2.4	3.1	8.9	1.2	7.2	11.3	81.2	7.5
A	Ci38	90.22	18.44	60.93	10.86	246.36	36.68	22.39	2.42	78.23	150.69	152.56	33.82	98.95	126.33	2.7	2.0	4.9	1.5	3.3	20.4	67.5	12.0
N	Ci39	58.17	15.78	35.44	6.94	226.48	35.92	17.26	1.86	77.00	148.32	152.19	33.74	99.04	126.21	3.9	2.3	3.7	1.6	2.2	27.1	60.9	11.9
G	Ci40	123.67	17.19	94.69	11.80	374.92	47.63	34.35	3.71	82.11	158.16	195.97	43.44	99.16	126.06	3.0	2.8	7.2	1.3	5.5	13.9	76.6	9.5

		**HPLC method**				**Spectrophotometric method**		**Antioxidant activity**						**α-Glucosidase inhibitory activity**		**HPLC results / Spectrophotometric results ratios**							
		SPP	SF	SET	SPA	TPC	TFC	ABTS		DPPH		FRAP				TPC/SPP	TFC/SF	SPP/SF	SPP/SET	SET/SF	SF in SPP	SET in SPP	SPA in SPP
**Origin**	**Extract number**	[mg/g d.w]				[mg GAE/g d.w.]	[mg ME/g d.w.]	[% inhibition]	[mM GAE/g]	[% inhibition]	[mM GAE/g]	[mM Fe]	[mM GAE]	[% inhibition]	[µg/mL]						[%]	[%]	[%]
T	Ci41	90.63	18.77	62.05	9.82	269.53	31.62	18.77	2.03	75.02	144.50	174.08	38.59	99.42	125.73	3.0	1.7	4.8	1.5	3.3	20.7	68.5	10.8
N	Ci42	87.00	13.13	66.10	7.78	225.08	29.54	23.54	2.54	74.41	143.33	186.51	41.34	96.96	128.92	2.6	2.3	6.6	1.3	5.0	15.1	76.0	8.9
A	Ci43	86.08	18.77	55.96	11.34	410.44	35.97	25.58	2.76	81.05	156.12	212.16	47.03	99.79	125.26	4.8	1.9	4.6	1.5	3.0	21.8	65.0	13.2
T	Ci44	104.50	17.05	77.87	9.58	392.49	36.04	15.99	1.73	76.01	146.41	223.92	49.63	99.28	125.91	3.8	2.1	6.1	1.3	4.6	16.3	74.5	9.2
T	Ci45	192.03	18.89	160.28	12.86	560.34	55.41	30.26	3.27	81.23	156.46	287.08	63.63	97.57	128.12	2.9	2.9	10.2	1.2	8.5	9.8	83.5	6.7
T	Ci46	55.35	22.89	27.79	4.68	13.62	26.46	15.76	1.70	75.68	145.78	187.51	41.56	94.67	132.04	0.2	1.2	2.4	2.0	1.2	41.4	50.2	8.4
N	Ci47	94.34	16.58	68.74	9.03	273.18	30.69	15.64	1.69	73.84	142.23	205.27	45.50	99.53	125.59	2.9	1.9	5.7	1.4	4.1	17.6	72.9	9.6
T	Ci48	73.96	14.54	52.83	6.59	210.85	23.20	10.28	1.11	71.12	137.00	163.78	36.30	99.11	126.12	2.9	1.6	5.1	1.4	3.6	19.7	71.4	8.9
T	Ci49	182.64	18.91	152.62	11.11	210.61	53.66	34.37	3.71	81.31	156.63	267.28	59.25	96.10	130.08	1.2	2.8	9.7	1.2	8.1	10.4	83.6	6.1
N	Ci50	65.44	12.13	47.32	5.98	128.97	21.45	7.04	0.76	68.95	132.82	162.20	35.95	97.73	127.90	2.0	1.8	5.4	1.4	3.9	18.5	72.3	9.1
T	Ci51	228.75	18.48	189.36	20.92	441.08	72.12	38.97	4.21	85.57	164.83	254.34	56.38	96.75	129.20	1.9	3.9	12.4	1.2	10.2	8.1	82.8	9.1
N	Ci52	156.35	18.98	127.40	9.96	414.16	40.36	37.33	4.03	77.21	148.73	213.80	47.39	98.32	127.14	2.6	2.1	8.2	1.2	6.7	12.1	81.5	6.4

A, Albania; G, Greece; T, Turkey; N, of unknown origin; g d.w., a gram of dry weight; SPP, sum of polyphenols; SF, sum of flavonoids; SET, sum of ellagitanins; SPA, sum of phenolic acids; TPC, total phenolic content; TFC, total flavonoid content; mg GAE, gallic acid equivalents; mg ME, myricetin equivalents.

S3. Ability of α-glucosidase inhibition by *C. incanus*

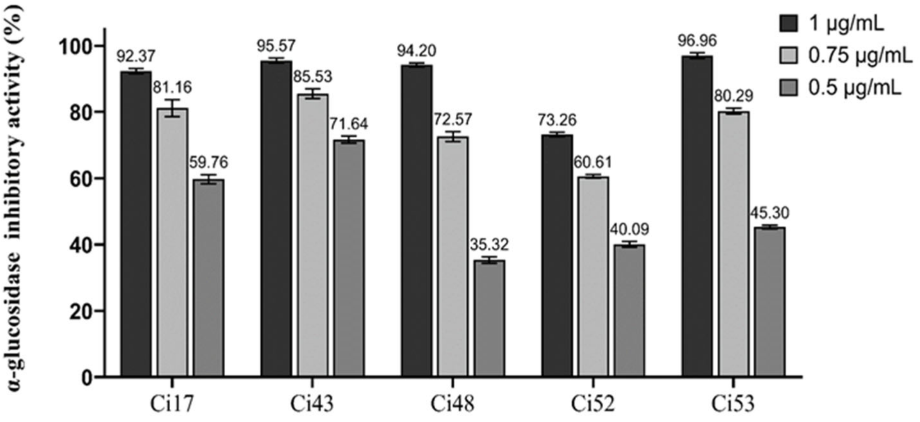

Figure S1. Inhibition of α-glucosidase by *C. incanus* extracts at different concentrations.

The absorbance values have been used to determine the changes in Michaelis constant (K_m) and maximum velocity of reaction (V_{max}). At first, the change in absorbance over time was converted to absorbance units per minute and then expressed as micromoles of chromogenic product (*p*-Np) liberated in the enzymatic reaction in order to determine reaction velocity. The molar extinction coefficient of *p*-NP used in the calculation was determined from its standard curve. The reciprocal of reaction velocity values of each tested compound and extract at different substrate concentrations were then plotted against the reciprocal of substrate concentrations. The intercepts of the created trendlines were utilized to calculate K_m and V_{max} values of each enzymatic reaction, and evaluation of alterations in the two parameters allowed us to determine the type of inhibition demonstrated by every compound.

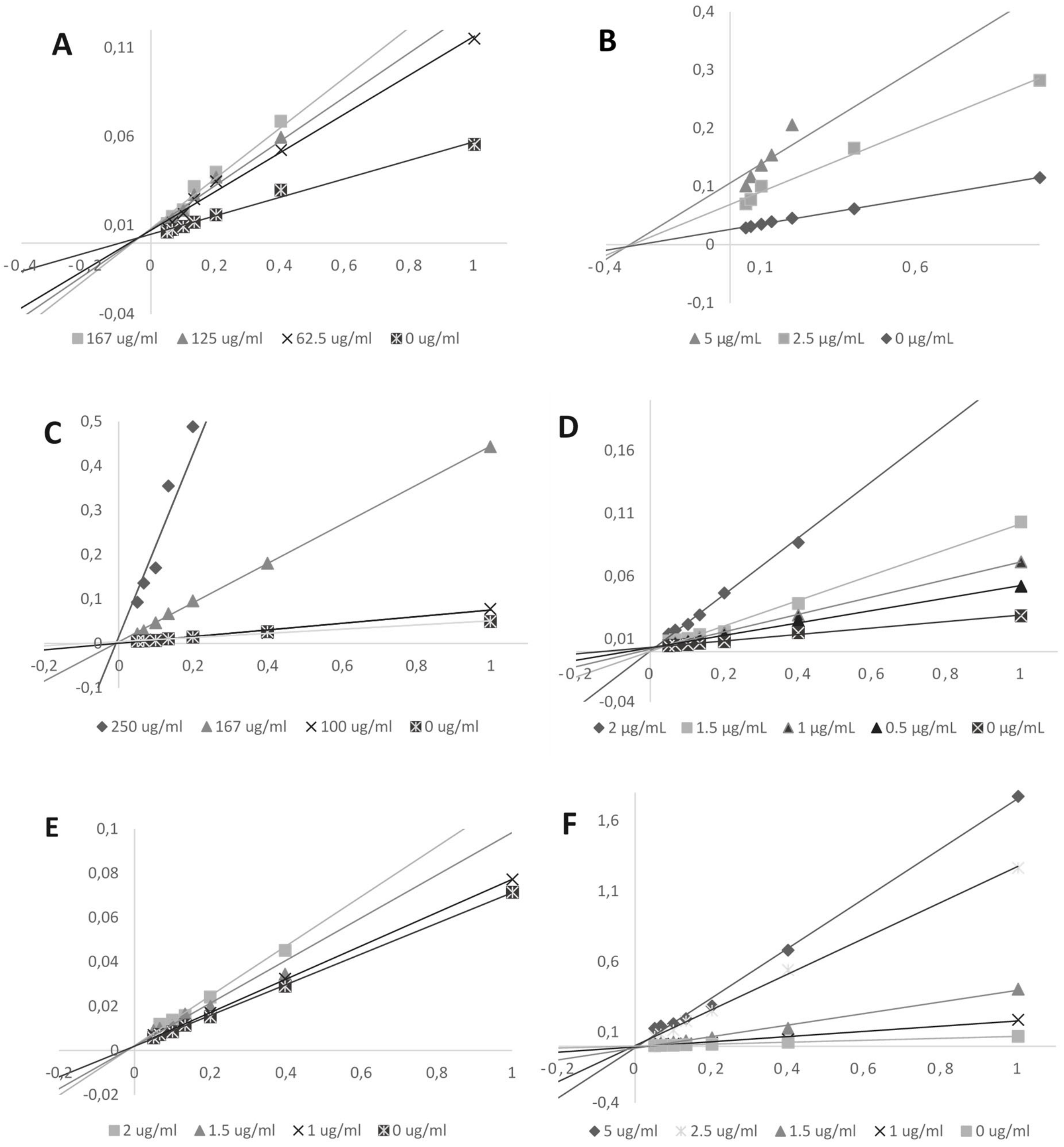

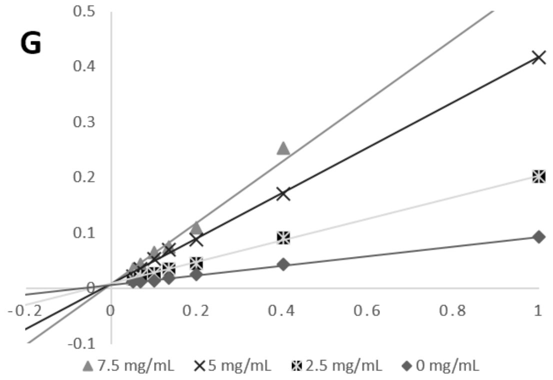

Figure S2. Lineweaver-Burk plots—the inverse initial velocity as a function of the inverse of the substrate concentration—with *p*-NPG as a substrate of α-glucosidase inhibitory activity of tiliroside (A), ellagic acid (B), gallic acid (C), terflavin A (D), punicalagin (E) and cistusin (F). Punicalagin (E) and acarbose (G) were determined to be competitive inhibitors of the enzyme. The others show a mixed type of inhibition. The x-axis is the reciprocal of substrate concentration (1/[S]), while the y-axis is the reciprocal of the reaction velocity (1/V).

S4. Statistical analysis

Table S9. Test of normality of distribution.

Variable	Origin	N	max. D	K-S	Lillief.	W	*p*-ratio
Sum of polyphenols (SPP)	A	10	0.118	p > .20	p > .20	0.942	0.573
Sum of ellagitannins (SET)	A	10	0.134	p > .20	p > .20	0.926	0.407
Sum of flavonoids (SF)	A	10	0.152	p > .20	p > .20	0.967	0.867
Sum of phenolic acids (SPA)	A	10	0.243	p > .20	p < .10	0.864	0.085
Total phenolic content (TPC)	A	10	0.231	p > .20	p < .15	0.917	0.332
Total flavonoid content (TFC)	A	10	0.119	p > .20	p > .20	0.967	0.859
Sum of polyphenols (SPP)	O	20	0.142	p > .20	p > .20	0.922	0.106
Sum of flavonoids (SF)	O	20	0.126	p > .20	p > .20	0.958	0.511
Sum of ellagitannins (SET)	O	20	0.138	p > .20	p > .20	0.932	0.165
Sum of phenolic acids (SPA)	O	20	0.205	p > .20	p < .05	0.860	0.008
Total phenolic content (TPC)	O	20	0.133	p > .20	p > .20	0.977	0.886
Total flavonoid content (TFC)	O	20	0.164	p > .20	p < .20	0.917	0.086
Sum of polyphenols (SPP)	T	22	0.187	p > .20	p < .05	0.918	0.069
Sum of flavonoids (SF)	T	22	0.128	p > .20	p > .20	0.978	0.883
Sum of ellagitannins (SET)	T	22	0.204	p > .20	p < .01	0.901	0.032
Sum of phenolic acids (SPA)	T	22	0.164	p > .20	p < .15	0.906	0.040
Total phenolic content (TPC)	T	22	0.091	p > .20	p > .20	0.976	0.835
Total flavonoid Content (TFC)	T	22	0.147	p > .20	p > .20	0.955	0.398

A, Albania; O, other countries including Greece; T, Turkey

Table S10. Statistical significance between the results of the mean contents of groups of components.

Variable	SS	df	MS	SS	df	MS	F	*p*-ratio
Sum of polyphenols (SPP)	18982.56	2	9491.279	67060.5	49	1368.58	6.935	0.002
Sum of ellagitannins (SET)	17158.36	2	8579.181	60637.0	49	1237.49	6.933	0.002
Sum of flavonoids (SF)	21.74	2	10.872	243.3	49	4.97	2.190	0.123
Sum of phenolic acids (SPA)	14.00	2	7.001	395.7	49	8.07	0.867	0.427
Total phenolic content (TPC)	5933.59	2	2966.797	954884.8	49	19487.44	0.152	0.859
Total flavonoid content (TFC)	1041.43	2	520.717	6365.9	49	129.92	4.008	0.024

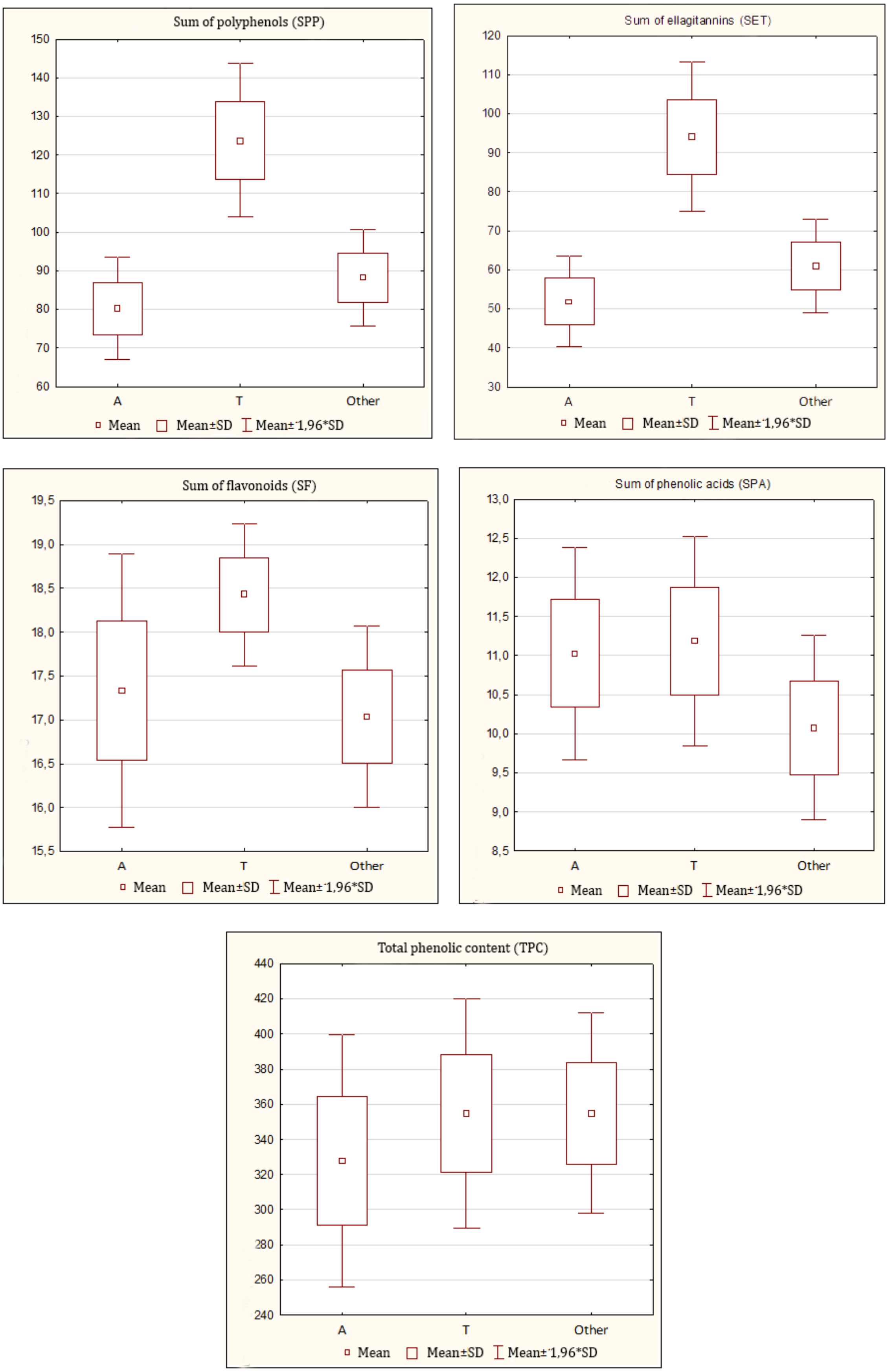

Figure S3. Groups of *C. incanus* components with statistically significant difference in contents.

Table S11. Fisher's LSD test for selected groups of components between countries.

Variable / Origin	Sum of polyphenols (SPP)			Sum of ellagitannins (SET)			Total flavonoid content (TFC)		
	Albania	Turkey	Other	Albania	Turkey	Other	Albania	Turkey	Other
Albania	—	0.003	0.582	—	0.003	0.503	—	0.013	0.382
Turkey	0.003	—	0.003	0.003	—	0.004	0.013	—	0.043
Other	0.582	0.003	—	0.503	0.004	—	0.382	0.043	—

Table S12. Normality of distribution for antioxidant assays.

Variable	Origin	N	max. D	K-S	Lillief.	W	*p*-ratio
ABTS	A	10	0.131	p > .20	p > .20	0.949	0.654
DPPH	A	10	0.221	p > .20	p < .20	0.943	0.587
FRAP	A	10	0.199	p > .20	p > .20	0.940	0.549
ABTS	O	20	0.123	p > .20	p > .20	0.953	0.407
DPPH	O	20	0.139	p > .20	p > .20	0.949	0.348
FRAP	O	20	0.117	p > .20	p > .20	0.954	0.439
ABTS	T	22	0.118	p > .20	p > .20	0.947	0.281
DPPH	T	22	0.153	p > .20	p < .20	0.938	0.178
FRAP	T	22	0.075	p > .20	p > .20	0.991	0.999

A, Albania; O, other countries including Greece; T, Turkey

Table S13. Analysis of variance (ANOVA) for antioxidant assays.

Variable	SS	df	MS	SS	df	MS	F	*p*-ratio
ABTS	3168.69	2	1584.34	11055.4	49	225.620	7.022	0.002
DPPH	76.83	2	38.41	1313.5	49	26.806	1.433	0.248
FRAP	27083.27	2	13541.64	108982.4	49	2224.131	6.089	0.004

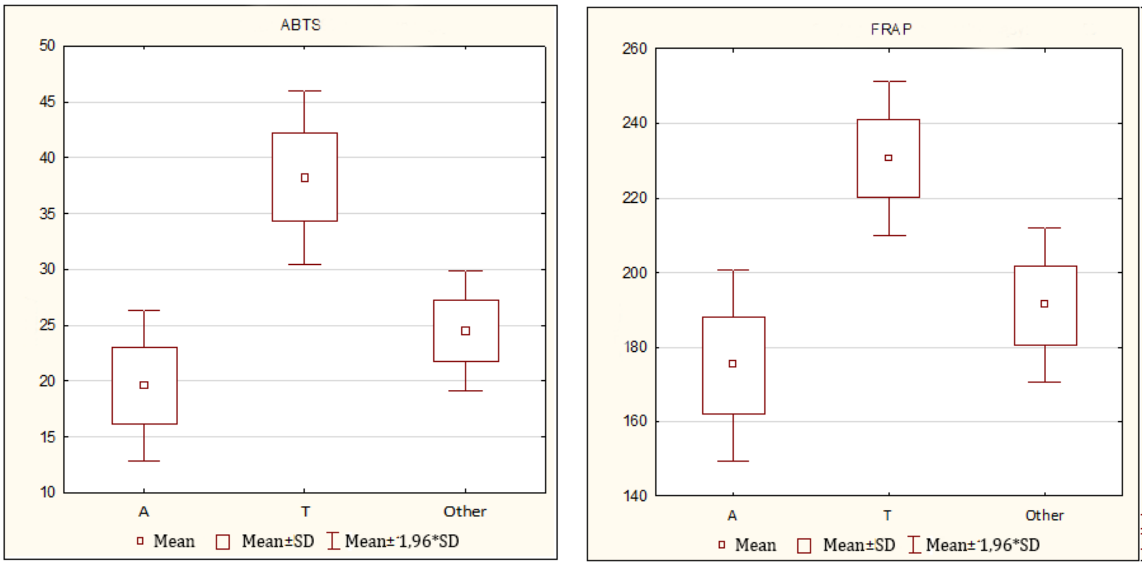

Figure S4. Antioxidant tests showing statistically significant differences.

Table S14. Fisher's LSD test for ABTS and FRAP tests between countries.

Origin \ Variable	ABTS			FRAP		
	Albania	Turkey	Other	Albania	Turkey	Other
Albania	—	0.002	0.403	—	0.003	0.383
Turkey	0.002	—	0.005	0.003	—	0.009
Other	0.403	0.005	—	0.383	0.009	—

Table S15. Linear regression and Spearman's rank correlation coefficient between component groups and antioxidant tests ABTS, DPPH, FRAP.

Test	Variables	Linear regression		Spearman's rank order correlation	
		R	*p*-ratio	R	*p*-ratio
ABTS	Sum of polyphenols (SPP)	0.41	0.003	0.47	0.000
	Sum of flavonoids (SF)	0.47	0.000	0.52	0.000
	Sum of ellagitannins (SET)	0.37	0.006	0.43	0.001
	Sum of phenolic acids (SPA)	0.34	0.014	0.41	0.002
	Total phenolic content (TPC)	0.37	0.007	0.38	0.006
	Total flavonoid content (TFC)	0.47	0.000	0.57	0.000
DPPH	Sum of polyphenols (SPP)	0.37	0.008	0.34	0.014
	Sum of flavonoids (SF)	0.31	0.024	0.28	0.043
	Sum of ellagitannins (SET)	0.33	0.016	0.31	0.025
	Sum of phenolic acids (SPA)	0.47	0.000	0.39	0.004
	Total phenolic content (TPC)	0.33	0.018	0.35	0.011
	Total flavonoid content (TFC)	0.62	0.000	0.57	0.000
FRAP	Sum of polyphenols (SPP)	0.68	0.000	0.69	0.000
	Sum of flavonoids (SF)	0.46	0.001	0.52	0.000
	Sum of ellagitannins (SET)	0.66	0.000	0.65	0.000
	Sum of phenolic acids (SPA)	0.45	0.001	0.46	0.001
	Total phenolic content (TPC)	0.51	0.000	0.55	0.000
	Total flavonoid content (TFC)	0.78	0.000	0.77	0.000

Table S16. Linear regression between sum of polyphenols (SPP) and sum of ellagitannins (SET) in products of different origin.

Variables	Linear regression		
	Origin	R	*p*-ratio
Sum of polyphenols (SPP) & Sum of ellagitannins (SET)	Albania	0.99	0.000
Sum of polyphenols (SPP) & Sum of ellagitannins (SET)	Other	0.99	0.000
Sum of polyphenols (SPP) & Sum of ellagitannins (SET)	Turkey	1.00	0.000

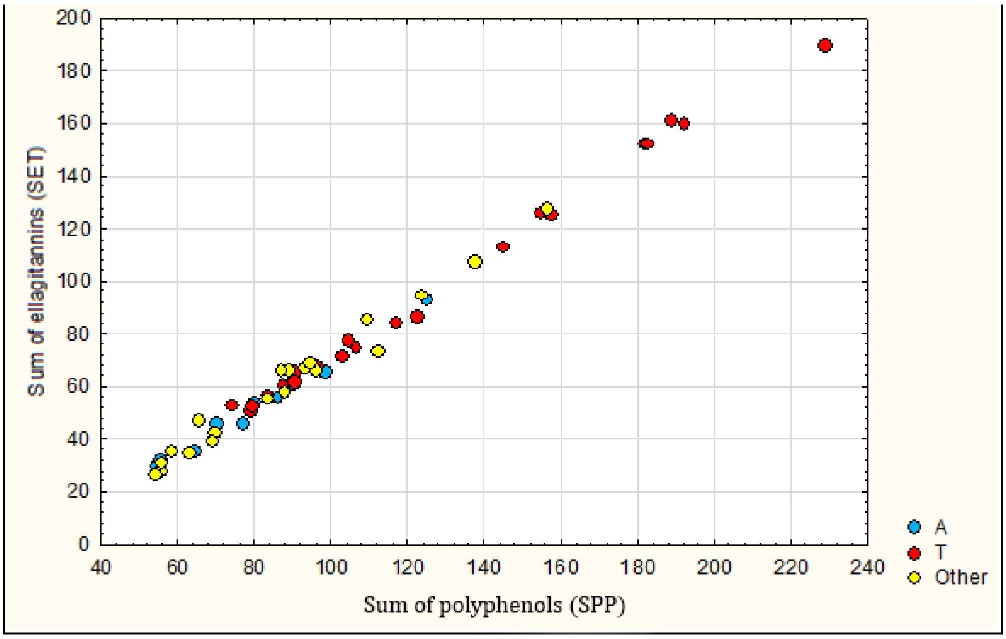

Figure S5. Correlation between sum of polyphenols (SPP) and sum of ellagitannins (SET) in products of different origin.
